# Semaglutide Treatment in Young Adults Living With Type 2 Diabetes: A Post Hoc Analysis From the SUSTAIN and PIONEER Clinical Trials

**DOI:** 10.1111/dom.70770

**Published:** 2026-04-17

**Authors:** Francesco Zaccardi, Vanita R. Aroda, Ecenur Guder Arslan, Lars Bardtrum, Jonathan Goldney, Erik Ising, Prachi Priyadarshini, Tommy Slater, Melanie J. Davies

**Affiliations:** ^1^ Leicester Real World Evidence Unit, Diabetes Research Centre University of Leicester Leicester UK; ^2^ Diabetes Research Centre University of Leicester Leicester UK; ^3^ Division of Endocrinology, Diabetes and Hypertension Brigham and Women's Hospital, Harvard Medical School Boston Massachusetts USA; ^4^ Novo Nordisk A/S Søborg Denmark; ^5^ NIHR Leicester Biomedical Research Centre University Hospitals of Leicester NHS Trust Leicester UK; ^6^ Novo Nordisk GBS Bengaluru India

**Keywords:** cohort study, GLP‐1, glycaemic control, meta‐analysis, semaglutide, type 2 diabetes

## Abstract

**Aims:**

Young adults (aged ≤ 40 years) are underrepresented in clinical trials that investigate interventions for those living with Type 2 diabetes (T2D). This study evaluated the efficacy of semaglutide treatment in young adults with T2D by examining the effects on HbA_1c_ and body weight (BW) during the SUSTAIN and PIONEER programmes compared to placebo and active comparators, according to age at study enrolment. This study also assessed aggregated safety data across age subgroups.

**Materials and Methods:**

This *post hoc* analysis of the SUSTAIN (once‐weekly subcutaneous administration) and PIONEER (once‐daily oral administration) programmes assessed the efficacy of semaglutide treatment in different age subgroups by comparing change in HbA_1c_ and BW between young adults with T2D (≤ 40 years), middle‐aged adults with T2D (> 40‐ ≤ 50 years), and middle older‐aged adults with T2D (> 50 years). Selected safety outcomes were assessed, focusing on serious adverse events (SAEs) and gastrointestinal SAEs from the programmes.

**Results:**

Findings indicate a reduction in HbA_1c_ levels and BW in participants across all age subgroups that were treated with semaglutide, particularly in young adults versus other age subgroups. The proportion of participants experiencing SAEs was overall comparable between semaglutide treatment and comparators across age subgroups and administration route.

**Conclusions:**

Semaglutide shows notable and consistent efficacy in reducing HbA_1c_ and BW across all age subgroups with T2D, including young adults. Effective glucose‐ and weight‐lowering interventions in people living with T2D at an earlier stage in life may reduce the high risk of future health complications associated with developing T2D as a younger adult.

## 1 Introduction

Type 2 diabetes (T2D) is a global health issue, which is increasing in prevalence across the world [1], due largely to increased urbanization, increased sedentary lifestyles, and increased rates of obesity [2, 3]. T2D has typically been studied within middle‐ (> 40 and ≤ 50 years) and middle older‐aged (> 50 years) cohorts [4], although the diagnosis of T2D among younger adults (aged ≤ 40 years), often referred to as early‐onset T2D (EOT2D), has been increasing globally [5]. This increased prevalence of EOT2D may be explained primarily by an increase in the rate of obesity observed in younger populations [6]. Because the definition of EOT2D refers specifically to the age at which an individual is diagnosed with T2D rather than their current age, it is still possible for adults in middle‐ to middle older‐aged cohorts to have EOT2D.

Although the mechanisms involved in the development of EOT2D are likely similar to those seen in the development of T2D in older age groups [5], an earlier onset of T2D is associated with a more aggressive phenotype with accelerated complications [7], and is characterised by increased insulin resistance and a more rapid dysfunction of β‐cell function at an earlier stage [6, 8, 9, 10, 11], which may be linked to increasing rates of obesity prevalence [6, 7], and ultimately results in earlier deterioration of glycaemic control [10, 12]. Compared to people with the onset of T2D during middle‐ and middle‐older ages, people with EOT2D present with increased body mass index (BMI), higher fasting glucose levels at the time of diagnosis, higher glycaemic levels, and higher glycaemic variability [13, 14]. Consequently, the development of EOT2D is associated with a greater risk of adverse outcomes, including a higher risk of cardiovascular disease, faster progression of microvascular complications, and premature mortality [15]. Moreover, individuals with EOT2D often have increased levels of depressive symptoms and T2D‐specific distress, with lower levels of self‐compassion [16]. This can make self‐management more difficult for individuals with EOT2D, further influencing health outcomes and quality of life [17, 18]. Despite this wide array of T2D‐associated complications, young individuals with T2D are underrepresented within clinical trials, often excluded entirely from drug and lifestyle intervention trials [4].

International guidelines recommend that T2D treatment is initiated as early as possible to prevent the development of T2D‐associated complications [19, 20, 21]. The American Diabetes Association (ADA) and the European Association for the Study of Diabetes (EASD) Consensus Report advocate for the use of glucagon‐like peptide‐1 receptor agonists (GLP‐1 RAs) in the treatment of T2D, because they have been shown to improve cardiovascular and renal outcomes, but also have very high glycaemic‐lowering and weight‐loss efficacy [19, 20]. Semaglutide, a GLP‐1 RA in the once‐weekly (OW) subcutaneous (s.c.) and once‐daily (OD) oral dose forms [22, 23, 24], has demonstrated significant reductions in both glycated haemoglobin A1c (HbA_1C_) and body weight (BW) among adults with T2D in clinical trials, including the SUSTAIN [25, 26, 27, 28, 29, 30, 31, 32, 33, 34, 35] and PIONEER [36, 37, 38, 39, 40] programmes. Moreover, SUSTAIN 6 [22] and the SOUL trial [24] showed that OW s.c. and oral semaglutide, respectively, reduce major adverse cardiovascular events in people with T2D versus placebo. However, these trials only included participants that were aged ≥ 50 years. To this point, the majority of clinical trials in T2D management including phase 3 programmes testing glucose‐lowering therapies and trials examining self‐management and lifestyle interventions include cohorts that consist predominantly (~95%) of middle‐ to middle older‐aged populations [4]. This impacts the availability of evidence‐based treatments for younger adults. Given that younger adults with T2D have a consistently greater exposure to hyperglycaemia, more than three‐fold increase in cumulative glycaemic exposure by the age of 75 years as compared to those diagnosed later in life [41], and a higher risk of complications [42], there is an urgent need for evidence‐based glucose‐lowering therapies targeting younger adults [43]. Recently, the SURPASS programme, which included 20% participants with EOT2D, found that the use of tirzepatide (a GLP‐1/glucagon‐dependent insulinotropic polypeptide (GIP) dual agonist) was beneficial in individuals with EOT2D, noting that there were improvements in HbA_1c_ levels, BW reduction, and cardiometabolic markers observed for individuals across all age subgroups in the tirzepatide arm, as compared to the placebo or comparator arm [44].

Therefore, the current study focuses on this underrepresented population of adults aged ≤ 40 years, by performing a *post hoc* analysis of the SUSTAIN and PIONEER programmes that investigated the efficacy of semaglutide (s.c. and oral, respectively) in adults with T2D. Whilst there is a lack of consensus on what constitutes a younger age group in the literature, with a range of definitions applied, an age of ≤ 40 years was used to define the younger cohort in the present study, based on this being the most common definition of EOT2D used pragmatically in previous research [15]. Hence, this study aims to compare the efficacy and safety of semaglutide across three distinct age subgroups (≤ 40, > 40–≤ 50 and > 50 years) by examining the effects on HbA_1c_ and BW in those receiving semaglutide treatment compared to placebo or active comparator treatments.

## 2 Methods

### 2.1 Trial Design for the SUSTAIN and PIONEER Programmes

The full trial designs for the SUSTAIN and PIONEER programmes have been published previously and are summarised in Tables S1 and S2. Briefly, the SUSTAIN 1, 2, 5, 9, and China multi‐regional clinical trial (MRCT) trials and the PIONEER 1, 4, and 8 trials were double‐blinded. Conversely, the SUSTAIN 3, 4, 7, 10, Japan Oral Anti‐Diabetic (OAD) combination, and Japan monotherapy trials and the PIONEER 2 and 7 trials were open‐label. The SUSTAIN trials investigated OW s.c. semaglutide treatment that was either 0.5 mg or 1.0 mg, while the PIONEER trials investigated OD oral semaglutide treatment at doses 3, 7, or 14 mg. Across the SUSTAIN and PIONEER programmes, semaglutide was tested in participants at different clinical stages of T2D, and each trial reflected this with specific inclusion and exclusion criteria for concomitant medications (Table S1).

While there were additional studies in the SUSTAIN and PIONEER programmes, data from these studies were not included in the current *post hoc* analysis, due to either the inclusion of too few participants from younger age subgroups or if a study lacked a non‐semaglutide comparator. Additionally, the cardiovascular outcome trials (SUSTAIN 6, PIONEER 6, and SOUL) were excluded as they only included members of the middle older‐aged cohort.

### 2.2 Participants

The full inclusion and exclusion criteria for the SUSTAIN and PIONEER programmes have been published previously and key criteria are summarised in Table S1, but were largely similar across trials. Participants were adults (aged ≥ 18 years or ≥ 20 years in Japan) with T2D and HbA_1c_ levels between 7.0% –10.0% (SUSTAIN 1, 4, 5 and 9), 7.0% –10.5% (SUSTAIN 2, 3, 7, Japan OAD combination, Japan Monotherapy, and China MRCT, and PIONEER 2), 7.0% –11.0% (SUSTAIN 10), or 7.0% –9.5% (PIONEER 1, 4, 7, and 8).

All trials were conducted in compliance with the Declaration of Helsinki and the International Conference on Harmonization Good Clinical Practice Guidelines. The protocols were approved by Independent Local Ethics Committees and Institutional Review Boards at each participating centre. Participants provided informed consent before the commencement of any study‐related activities. Clinical trial identification numbers can be found in Table S2.

### 2.3 Age Subgroups

Participants across the SUSTAIN and PIONEER programmes were stratified into subgroups (*post hoc*) based on their age at study enrolment: (1) ≤ 40 years (younger‐aged T2D), (2) > 40–≤ 50 years (middle‐aged T2D), and (3) > 50 years (middle older‐aged T2D). Importantly, the age at study enrolment was used to categorise participants. Data were evaluated for each trial separately, and *post hoc* exploratory analyses were performed to compare outcomes for all participants stratified by age subgroup.

### 2.4 Endpoints

The primary endpoint for all the SUSTAIN and PIONEER trials was change in HbA_1C_ from baseline, while the confirmatory secondary endpoint was change in BW from baseline (except for SUSTAIN Japan monotherapy and Japan OAD combination, whose primary endpoint was safety, and supportive secondary efficacy endpoints included change in HbA_1c_ and change in BW, and PIONEER 7, whose primary endpoint was whether or not a participant achieved HbA_1c_ < 7%). Primary endpoints were either assessed at week 26, 30, 40, or 56 (further details can be found in Tables S1 and S2). For this *post hoc* analysis, both HbA_1c_ and BW were stratified by age subgroups and analysed separately for each study.

### 2.5 Safety

The safety assessment included the percentage and rate of participants experiencing serious adverse events (SAEs), with gastrointestinal (GI) SAEs described specifically, as well as GI adverse events (AEs), across both the SUSTAIN and PIONEER programmes. The occurrence of all SAEs and GI AEs was pooled into four categories: s.c. semaglutide treatment versus comparators for the SUSTAIN trials, and oral semaglutide versus comparators for the PIONEER trials. Included in the total GI AEs, the percentage and rate of participants experiencing GI AEs leading to discontinuation was also reported. The percentages and rates for both SAEs and GI AEs were pooled into their respective categories across studies and corrected using a Cochrane‐Mantel–Haenszel adjustment [45].

### 2.6 Statistical Analysis

Endpoints were analysed using a mixed model for repeated measures (MMRM), with treatment, age subgroup, sex, baseline value, and treatment by age subgroup interaction as covariates. All covariates were nested within each visit. The interaction *p* value for treatment by age was evaluated at the end of trial, and a value of *p* < 0.05 was considered statistically significant.

## 3 Results

### 3.1 Baseline Characteristics

Key baseline characteristics, stratified by age subgroup, can be found for both programmes in Table 1. This *post hoc* analysis included 11 223 total participants, with 7753 participants belonging to the SUSTAIN programme (4470 on semaglutide), and 3470 participants belonging to the PIONEER programme (2983 on semaglutide). There were 628 participants (8.1%) in the younger‐aged subgroup, 1584 participants (20.4%) in the middle‐aged subgroup, and 5541 participants (71.5%) in the middle older‐aged subgroup for the SUSTAIN trials, and 204 participants (5.9%) in the younger‐aged subgroup, 672 (19.4%) participants in the middle‐aged subgroup, and 2594 (74.8%) participants in the middle older‐aged subgroup for the PIONEER trials (percentages may add to over 100% due to rounding).

**TABLE 1:** Baseline characteristics.

SUSTAIN trials: Once‐weekly subcutaneous											
Trial	Comparator	Age subgroup (years)	Age, mean (SD) years	HbA_1c_, mean (SD) %	Body weight, mean (SD) kg	BMI, mean (SD) kg/m^2^	Duration of diabetes, mean (SD) years	*N* total	*N* comparator	*N* sema‐glutide 0.5 mg	*N* sema‐glutide 1.0 mg
SUSTAIN 1	Placebo	≤ 40	35.0 (4.4)	8.2 (0.9)	103.5 (24.9)	37.1 (9.4)	1.8 (2.5)	50	13	13	24
		> 40–≤ 50	46.1 (2.6)	8.1 (0.9)	96.6 (24.4)	34.5 (7.6)	3.8 (4.5)	104	37	38	29
		> 50	61.1 (7.0)	8.0 (0.8)	87.4 (22.1)	31.3 (6.8)	4.8 (6.2)	233	79	77	77
SUSTAIN 2	Sitagliptin 100 mg	≤ 40	35.7 (3.9)	8.4 (0.9)	96.4 (23.4)	33.6 (6.7)	3.8 (3.0)	95	38	35	22
		> 40–≤ 50	46.3 (2.7)	8.2 (0.9)	92.9 (22.1)	33.2 (6.9)	5.2 (4.0)	300	101	106	93
		> 50	60.5 (6.5)	8.0 (0.9)	87.5 (18.8)	32.1 (5.9)	7.4 (5.4)	830	268	268	294
SUSTAIN 3	Exenatide extended release	≤ 40	34.6 (5.5)	8.6 (1.0)	107.9 (27.9)	38.3 (9.3)	5.6 (4.5)	61	29	—	32
		> 40–≤ 50	46.5 (2.9)	8.6 (1.0)	99.1 (22.1)	34.5 (7.2)	6.9 (4.7)	164	87	—	77
		> 50	61.6 (7.0)	8.3 (0.9)	93.6 (20.0)	33.1 (6.1)	10.2 (6.6)	584	289	—	295
SUSTAIN 4	Insulin glargine	≤ 40	35.1 (4.8)	8.4 (0.9)	101.1 (26.8)	35.4 (7.6)	6.1 (5.5)	74	26	26	22
		> 40–≤ 50	46.3 (2.8)	8.3 (1.0)	96.3 (24.5)	33.9 (7.0)	6.9 (5.4)	227	76	71	80
		> 50	61.4 (6.9)	8.1 (0.9)	91.9 (20.2)	32.5 (6.1)	9.3 (6.5)	781	258	265	258
SUSTAIN 5	Placebo	≤ 40	35.3 (5.2)	8.6 (0.8)	94.1 (16.6)	34.1 (6.2)	8.2 (5.0)	21	7	7	7
		> 40–≤ 50	46.2 (3.0)	8.6 (0.9)	94.8 (23.2)	32.9 (6.7)	8.2 (5.0)	48	21	13	14
		> 50	62.1 (7.1)	8.3 (0.8)	91.1 (20.9)	32.0 (6.1)	14.4 (7.8)	327	105	112	110
SUSTAIN 7	Dulaglutide 0.75 mg/Dulaglutide 1.5 mg	≤ 40	35.3 (4.2)	8.5 (1.0)	97.4 (30.8)	34.7 (8.8)	4.5 (4.4)	106	27/28	24	27
		> 40–≤ 50	46.0 (2.9)	8.3 (0.9)	98.2 (24.8)	34.6 (7.3)	5.6 (4.7)	257	70/61	68	58
		> 50	61.2 (6.7)	8.2 (0.9)	94.1 (20.4)	33.0 (6.2)	8.3 (5.8)	836	202/210	209	215
SUSTAIN 9	Placebo	≤ 40	35.1 (5.0)	8.4 (1.0)	94.2 (15.5)	33.0 (4.3)	5.9 (5.5)	19	11	—	8
		> 40–≤ 50	46.8 (2.7)	8.0 (0.9)	97.7 (22.3)	33.0 (5.9)	7.8 (6.1)	44	25	—	19
		> 50	60.6 (6.4)	8.0 (0.8)	90.4 (21.0)	31.7 (6.8)	10.3 (6.0)	239	115	—	124
SUSTAIN 10	Liraglutide 1.2 mg	≤ 40	36.3 (3.0)	8.2 (0.9)	115.0 (25.7)	38.9 (8.0)	4.2 (3.2)	29	15	—	14
		> 40–≤ 50	46.7 (2.6)	8.5 (1.1)	108.3 (23.1)	36.8 (7.5)	6.0 (4.5)	86	41	—	45
		> 50	63.4 (7.0)	8.2 (0.9)	93.6 (19.3)	32.8 (6.3)	10.2 (5.9)	462	231	—	231
SUSTAIN Japan OAD combination	OAD monotherapy + diet/exercise	≤ 40	35.4 (4.4)	8.5 (1.0)	92.0 (19.0)	31.6 (5.7)	4.7 (4.7)	31	6	12	13
		> 40–≤ 50	46.1 (2.8)	8.4 (1.0)	82.7 (16.6)	29.5 (5.3)	5.6 (3.7)	100	18	48	34
		> 50	62.7 (7.0)	8.0 (0.9)	67.8 (12.5)	25.4 (3.9)	9.8 (6.6)	469	96	179	194
SUSTAIN Japan monotherapy	Sitagliptin 100 mg	≤ 40	35.3 (5.3)	8.1 (0.7)	85.3 (23.4)	31.3 (7.2)	5.5 (6.5)	14	3	5	6
		> 40–≤ 50	45.9 (2.6)	8.4 (1.2)	76.7 (15.6)	27.3 (5.1)	5.9 (4.0)	61	21	16	24
		> 50	62.8 (7.5)	8.1 (0.9)	66.4 (11.0)	24.6 (3.3)	8.7 (6.6)	233	79	82	72
SUSTAIN—China MRCT	Sitagliptin 100 mg	≤ 40	35.0 (4.7)	8.3 (1.0)	86.1 (18.9)	30.1 (5.4)	3.7 (4.1)	128	43	46	39
		> 40–≤ 50	46.0 (2.9)	8.2 (0.9)	79.6 (14.5)	28.4 (4.9)	4.9 (3.9)	193	64	58	71
		> 50	59.7 (6.2)	8.0 (0.8)	73.0 (14.2)	27.1 (4.6)	7.5 (5.4)	547	183	184	180

PIONEER trials: Once‐daily oral													
Trial	Comparator	Age subgroup (years)	Age, mean (SD) years	HbA_1c_, mean (SD) %	Body weight, mean (SD) kg	BMI, mean (SD) kg/m^2^	Duration of diabetes, mean (SD) years	*N* total	*N* comparator	*N* sema‐glutide 3 mg	*N* sema‐glutide 7 mg	*N* sema‐glutide 14 mg	*N* sema‐glutide flex
PIONEER 1	Placebo	≤ 40	34.9 (4.2)	8.0 (0.6)	103.8 (24.5)	36.2 (7.2)	1.7 (2.4)	72	15	19	22	16	—
		> 40–≤ 50	46.0 (2.9)	8.0 (0.7)	92.3 (22.3)	33.1 (6.7)	2.3 (2.9)	182	56	44	33	49	—
		> 50	61.2 (6.8)	7.9 (0.7)	83.9 (20.0)	30.6 (6.0)	4.4 (5.6)	449	107	112	120	110	—
PIONEER 2	Empagliflozin 25 mg	≤ 40	36.3 (3.7)	8.4 (1.1)	99.8 (26.4)	34.7 (7.4)	4.1 (2.9)	43	21	—	—	22	—
		> 40–≤ 50	45.9 (2.9)	8.3 (1.0)	96.7 (20.5)	34.2 (7.1)	4.4 (3.2)	147	70	—	—	77	—
		> 50	61.8 (6.7)	8.1 (0.9)	89.9 (19.4)	32.4 (5.7)	8.4 (6.4)	631	319	—	—	312	—
PIONEER 4	Liraglutide 1.8 mg/Placebo	≤ 40	37.1 (3.5)	8.3 (0.6)	102.9 (20.0)	34.5 (6.1)	5.6 (3.0)	43	21/6	—	—	16	—
		> 40–≤ 50	46.3 (2.6)	7.9 (0.7)	97.4 (21.9)	33.9 (6.9)	5.7 (4.3)	162	62/35	—	—	65	—
		> 50	61.2 (6.5)	7.9 (0.7)	92.1 (20.5)	32.5 (6.0)	8.4 (5.9)	506	201/101	—	—	204	—
PIONEER 7	Sitagliptin 100 mg	≤ 40	35.5 (5.2)	8.3 (0.7)	93.9 (20.4)	33.3 (5.3)	3.9 (2.7)	26	11	—	—	—	15
		> 40–≤ 50	46.8 (2.8)	8.3 (0.6)	94.4 (22.8)	34.0 (7.0)	5.9 (3.9)	90	43	—	—	—	47
		> 50	61.3 (7.1)	8.3 (0.6)	87.0 (18.8)	30.8 (6.0)	9.8 (6.5)	388	197	—	—	—	191
PIONEER 8	Placebo	≤ 40	35.4 (5.1)	8.4 (1.0)	94.0 (24.4)	34.1 (7.8)	7.4 (3.9)	20	7	6	4	3	—
		> 40–≤ 50	46.4 (2.5)	8.3 (0.8)	88.8 (21.7)	31.7 (6.2)	11.3 (6.6)	91	21	21	23	26	—
		> 50	63.6 (7.0)	8.2 (0.7)	85.2 (21.7)	30.8 (6.7)	15.8 (8.1)	620	156	157	155	152	—

*Note*: Data are presented as mean (SD).

Abbreviations: BMI, body mass index; HbA_1c_, glycated haemoglobin A1c; *N*, number of participants contributing to the analysis (defined as number of participants with a non‐missing baseline value, non‐missing factors and having at least one post‐baseline measurement of the endpoint); SD, standard deviation.

Across all age subgroups in both programmes, baseline HbA_1c_ levels were comparable across trials. Baseline BW (and BMI) were overall higher in the younger‐aged adults compared to middle‐aged and middle older‐aged adults (Table 1).

### 3.2 Change in HbA_1c_ From Baseline

#### 3.2.1 Estimated Treatment Difference (ETD) for Semaglutide in the Younger‐Aged Subgroup in HbA_1c_ Across Both SUSTAIN and PIONEER Programmes

**FIGURE 1:**
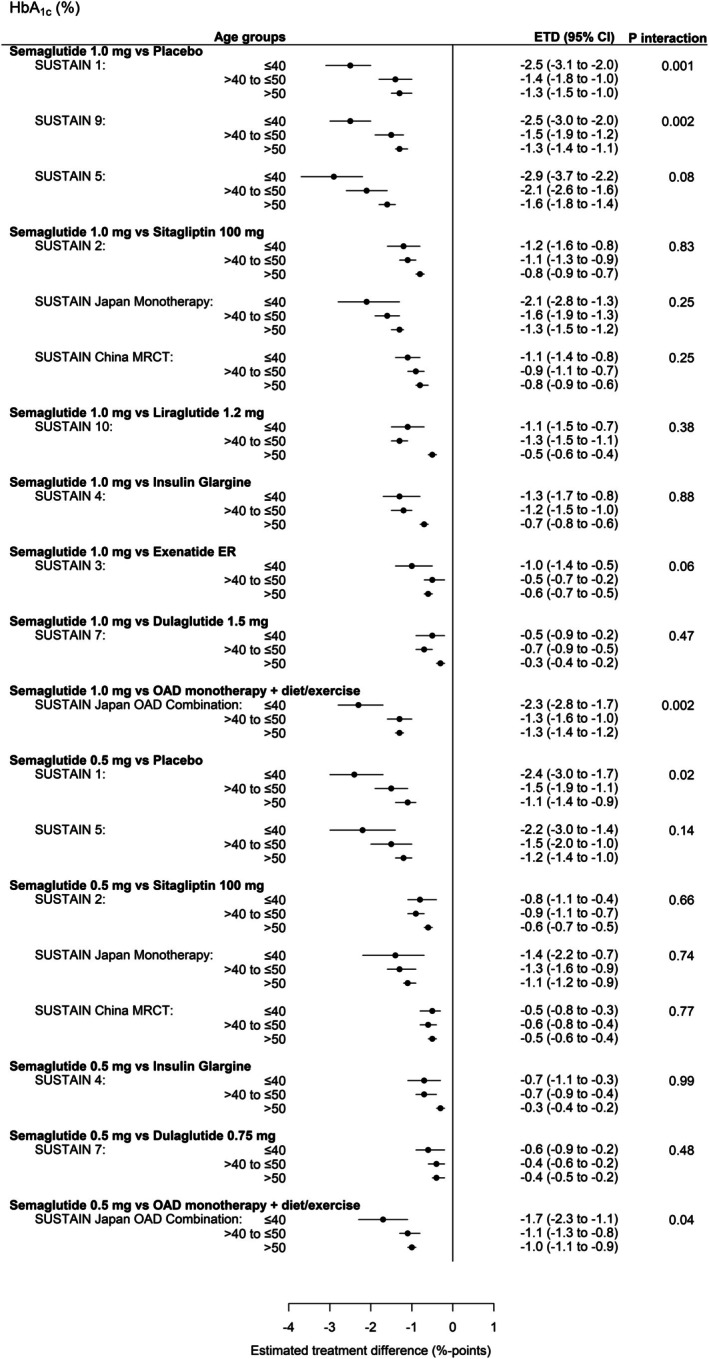
Change in HbA_1c_ from baseline in the SUSTAIN trials. Data are shown with estimated treatment difference (95% confidence interval) for each age subgroup. P interaction value represents the *p* value for the interaction between age subgroups and treatment arms. Abbreviations: CI, confidence interval; ETD, estimated treatment difference; ER, extended release; HbA_1c_, glycated haemoglobin A1c; MRCT, multi‐regional clinical trial; OAD, oral anti‐diabetic.

**FIGURE 2:**
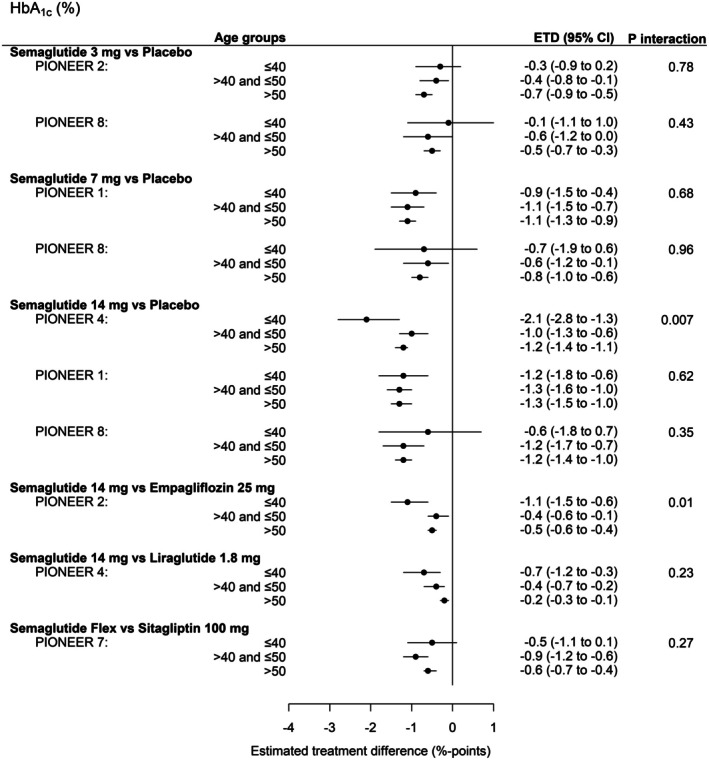
Change in HbA_1c_ from baseline in the PIONEER trials. Data are shown with estimated treatment difference (95% confidence interval) for each age subgroup. P interaction value represents the *p* value for the interaction between age subgroups and treatment arms. Abbreviations: CI, confidence interval; ETD, estimated treatment difference; HbA_1c_, glycated haemoglobin A1c.

The ETD in the change in HbA_1c_ %‐points for semaglutide versus comparators from baseline is shown in Figure 1 for the SUSTAIN trials and in Figure 2 for the PIONEER trials, while the estimated absolute changes in HbA_1c_ (%) for these programmes are provided in Table S3. The HbA_1c_ endpoints are also summarised in Table S4. In both programmes, there was a consistent effect across all age subgroups for semaglutide versus comparators. When comparing s.c. semaglutide versus placebo, the ETD (95% CI) for the younger‐adult subgroup ranged from −2.4% (−3.0, −1.7) in SUSTAIN 1 to −2.2% (−3.0, −1.4) in SUSTAIN 5 for 0.5 mg semaglutide treatment and −2.9% (−3.7, −2.2) in SUSTAIN 5 to −2.5% (−3.0, −2.0) in SUSTAIN 9 for 1.0 mg semaglutide treatment. As compared to an active comparator, the ETD (95% CI) for s.c. semaglutide ranged from −1.7% (−2.3, −1.1) versus OAD monotherapy + diet/exercise in SUSTAIN Japan OAD Combination to −0.5% (−0.8, −0.3) versus sitagliptin 100 mg in SUSTAIN China MRCT for 0.5 mg semaglutide treatment, and from −2.3% (−2.8, −1.7) versus OAD monotherapy + diet/exercise in SUSTAIN Japan Combination to −0.5% (−0.9, −0.2) versus dulaglutide 1.5 mg in SUSTAIN 7 for 1.0 mg semaglutide treatment. Comparing oral semaglutide to placebo, the ETD for the young‐adult subgroup ranged from −2.1% (−2.8, −1.3) in PIONEER 4 (14 mg) to −0.6% (−1.8, 0.7) in PIONEER 8 (14 mg). As compared to an active comparator, the ETD ranged from −1.1% (−1.5, 0.6) versus empagliflozin 25 mg in PIONEER 2 (14 mg) to −0.5% (−1.1, 0.1) versus sitagliptin 100 mg in PIONEER 7 (flex). For the PIONEER programme, the ETD for 3 mg semaglutide treatment was not taken into consideration because it was a starting dose.

The treatment effect for the change in HbA_1c_ %‐points from baseline with semaglutide versus comparators was significantly greater in the younger‐aged subgroup as compared to the middle‐ and middle older‐aged subgroups across a number of SUSTAIN and PIONEER trials (Figures 1 and 2, respectively). Compared to placebo, there was a greater treatment effect for younger adults versus middle older‐age subgroups in SUSTAIN 1 semaglutide 0.5 mg (*p*_interaction_ [*p*_int_] = 0.02) and semaglutide 1.0 mg (*p*_int_ = 0.001), SUSTAIN 9 semaglutide 1.0 mg (*p*_*i*nt_ = 0.002), and PIONEER 4 semaglutide 14 mg (*p*_int_ = 0.007). Compared to OAD monotherapy + diet/exercise in SUSTAIN Japan OAD combination, the greatest treatment effect was seen in young adults treated with semaglutide 0.5 mg (*p*_int_ = 0.04) and semaglutide 1.0 mg (*p*_int_ = 0.002). Similarly, in comparison to empagliflozin 25 mg, there was a greater treatment effect for younger adults in PIONEER 2 semaglutide 14 mg (*p*_int_ = 0.01) as compared to middle older‐age subgroups.

### 3.3 Change in Body Weight From Baseline

#### 3.3.1 ETD for Semaglutide in the Younger‐Aged Subgroup in BW Across Both SUSTAIN and PIONEER Programmes

**FIGURE 3:**
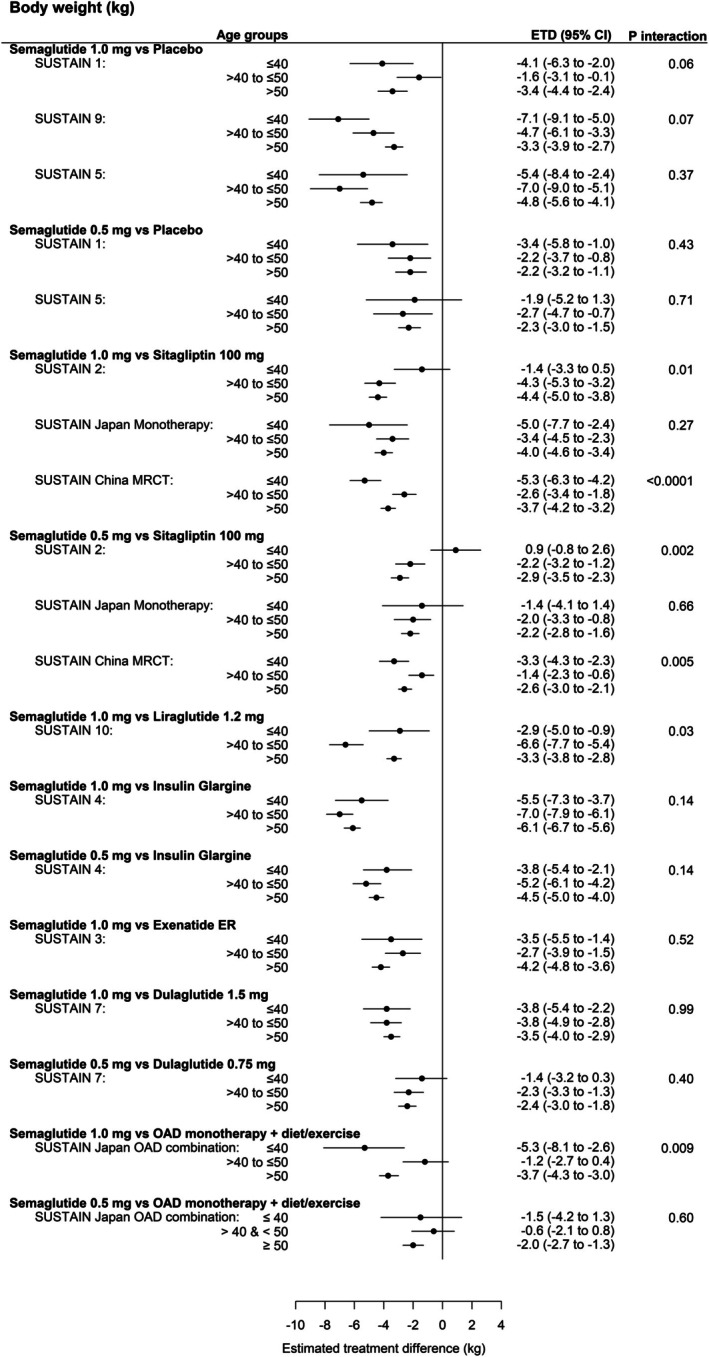
Change in body weight from baseline in the SUSTAIN trials. Data are shown with estimated treatment difference (95% confidence interval) for each age subgroup. P interaction value represents the *p* value for the interaction between age subgroups and treatment arms. Abbreviations: CI, confidence interval; ETD, estimated treatment difference; ER, extended release; MRCT, multi‐regional clinical trial; OAD, oral anti‐diabetic.

**FIGURE 4:**
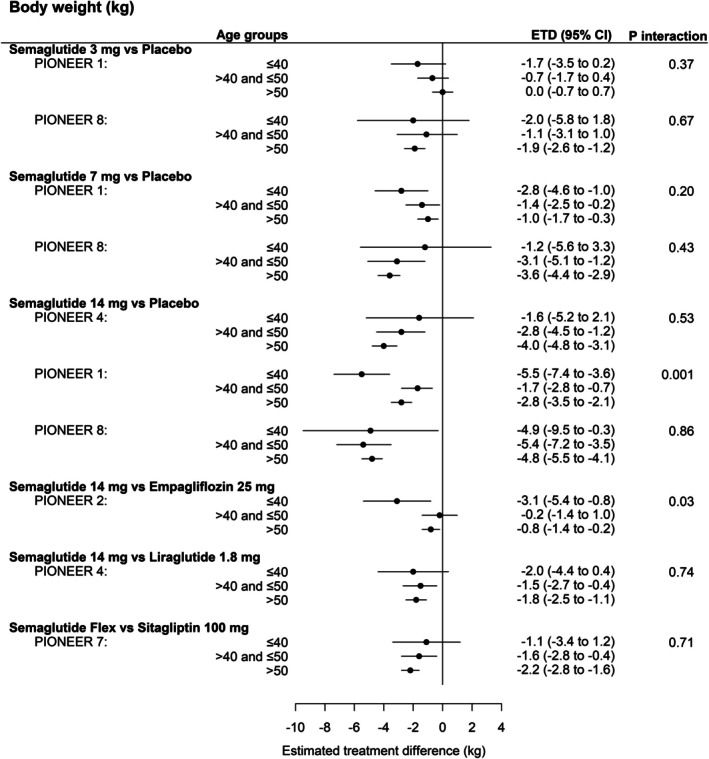
Change in body weight from baseline in the PIONEER trials. Data are shown with estimated treatment difference (95% confidence interval) for each age subgroup. P interaction value represents the *p* value for the interaction between age subgroups and treatment arms. Abbreviations: CI, confidence interval; ETD, estimated treatment difference.

The ETD in the change in BW (kg) from baseline can be seen in Figure 3 for the SUSTAIN trials and Figure 4 for the PIONEER trials, while the estimated absolute change in BW (kg) for these programmes is provided in Table S5. The BW endpoints are also summarised in Table S6.

There was a consistent treatment effect for change in BW with semaglutide versus comparators in all age subgroups across both programmes, with exception of SUSTAIN 2 semaglutide 0.5 mg versus sitagliptin 100 mg, where the observed ETD was numerically positive in the younger‐adult subgroup. In the young‐adult subgroup versus placebo, the ETD (95% CI) for s.c. semaglutide 0.5 mg ranged from −3.4 kg (−5.8, −1.0) in SUSTAIN 1 to −1.9 kg (−5.2, 1.3) in SUSTAIN 5; for s.c. semaglutide 1.0 mg, it ranged from −7.1 kg (−9.1, −5.0) in SUSTAIN 9 to −4.1 kg (−6.3, −2.0) in SUSTAIN 1. Compared to an active comparator, the ETD (95% CI) for 0.5 mg and 1.0 mg semaglutide treatment in the young‐adult subgroup ranged from −3.8 kg (−5.4, −2.1) in SUSTAIN 4 versus insulin glargine to +0.9 kg (−0.8, 2.6) in SUSTAIN 2 versus sitagliptin 100 mg, and from −5.5 kg (−7.3 to −3.7) in SUSTAIN 4 versus insulin glargine to −1.4 kg (−3.3, 0.5) in SUSTAIN 2 versus sitagliptin 100 mg, respectively. Similarly, compared to placebo, the ETD in the young‐adult subgroup for oral semaglutide ranged from −5.5 kg (−7.4, −3.6) in PIONEER 1 (14 mg) to −1.2 kg (−5.6, 3.3) in PIONEER 8 (7 mg). Compared to an active comparator, the ETD ranged from −3.1 kg (−5.4, −0.8) in PIONEER 2 (14 mg) versus empagliflozin 25 mg to −1.1 kg (−3.4, 1.2) in PIONEER 7 (flex) versus sitagliptin 100 mg. Overall, the ETD for BW reduction observed in the younger‐aged subgroup was comparable between SUSTAIN and PIONEER programmes.

The treatment effect for the change in BW from baseline with semaglutide versus comparators was significantly greater in the younger‐aged subgroup as compared to the middle‐ and middle older‐aged subgroups across a number of SUSTAIN and PIONEER trials (Figures 3 and 4, respectively). Compared to sitagliptin 100 mg, there was a greater treatment effect in the younger‐adult subgroup compared to older cohorts in SUSTAIN China MRCT semaglutide 0.5 mg (*p*_int_ = 0.005) and semaglutide 1.0 mg (*p*_int_ < 0.0001). For SUSTAIN Japan OAD combination, there was a greater treatment effect in younger adults compared to middle‐ and middle older‐aged adults (*p*_int_ = 0.009) for semaglutide 1.0 mg versus OAD monotherapy + diet/exercise. Compared to placebo, there was a greater treatment effect in the younger adults in PIONEER 1 semaglutide 14 mg (*p*_int_ = 0.001) than in middle‐ and middle older‐aged adults. Compared to empagliflozin 25 mg, there was a greater treatment effect in younger adults in PIONEER 2 semaglutide 14 mg (*p*_int_ = 0.03). However, compared to sitagliptin 100 mg, there was a lesser treatment effect in younger adults in SUSTAIN 2 semaglutide 0.5 mg (*p*_int_ = 0.002) and semaglutide 1.0 mg (*p*_int_ = 0.01) than middle‐ and middle older‐aged adults on semaglutide treatment.

### 3.4 Safety

#### 3.4.1 Serious Adverse Events

The number of participants with events, percentages and incidence rates of SAEs and GI SAEs by age subgroup are provided in Tables S7 and S8, respectively.

Overall, the proportion of participants with SAEs was similar between semaglutide and comparators across age subgroups and routes of administration. Among younger adults with T2D receiving OW s.c. semaglutide versus comparators, the SAE event rate was higher with OW s.c. semaglutide, whereas the proportion of participants experiencing at least one SAE was similar between subgroups. The imbalance in event rate is due to a few participants on semaglutide experiencing more AEs reported in the same case (i.e., several symptoms reported). No clustering of Preferred Terms was identified during analysis. Importantly, no safety concerns were identified. Overall, there was a low number of participants experiencing SAEs, and no imbalance on the proportion of participants experiencing SAEs was observed.

#### 3.4.2 GI Adverse Events

GI AEs events and GI AEs events leading to treatment discontinuation are presented as the number of participants, percentages, and incidence rates by age subgroup for semaglutide s.c. and oral semaglutide in Tables S9 and S10, respectively.

Overall, the proportion of participants experiencing GI AEs in the semaglutide arms was comparable across age groups and routes of administration. A numerically lower proportion of participants in the younger‐aged cohort discontinued semaglutide due to GI AEs in comparison to other age subgroups across both programmes.

## 4 Discussion

In this *post hoc* analysis, we compared the efficacy of semaglutide treatment against placebo and active comparators in people living with T2D across the SUSTAIN and PIONEER programmes, with a stratification of participants according to age at study enrolment. The present study demonstrates that not only is semaglutide efficacious in reducing HbA_1c_ levels and BW in younger adults with T2D, but that the treatment effect for HbA_1C_ may be greater than in middle‐aged and middle older‐aged adults with T2D.

The rising global incidence of T2D in young adults, coupled with elevated lifetime risks of complications and mortality [4, 15], underscores the need for effective, evidence‐based interventions in this population. Historically, younger adults have been underrepresented in clinical trials, and few studies have reported intervention efficacy in this group [46, 47, 48]. In this *post hoc* analysis, semaglutide demonstrated clinically meaningful efficacy on glycaemic levels and BW in younger adults with T2D. GLP‐1 RAs have demonstrated reduced cardio‐kidney‐metabolic risk in individuals with EOT2D, and semaglutide has shown benefits beyond glycaemia, including weight reduction and favourable cardiovascular and kidney outcomes [22, 24, 49, 50]. Emerging data has shown that semaglutide improves functional and vascular outcomes in people with peripheral artery disease and T2D, highlighting potential benefits beyond traditional cardiometabolic endpoints [51]. Exploratory data also suggest improvements in risk factors such as fatty acid metabolism and inflammation [52, 53]. Evaluating the effects of semaglutide in younger cohorts is therefore pertinent, as early improvements in HbA_1c_ and BW may have durable implications for preventing multimorbidity.

While it is generally accepted that reducing HbA_1c_ and BW can delay the onset of T2D‐associated complications, few studies have investigated the effect of long‐term T2D management. The UK Prospective Diabetes Study (UKPDS), however, followed people with T2D from 1977 to 1991 to investigate the effects of early implementation of intensive T2D treatment, and found that early intensive treatment substantially decreased the risk of microvascular complications and long‐term risk of cardiovascular outcomes [54, 55, 56].

The findings from the current study should be interpreted in the context of some limitations. This study was a *post hoc* analysis of a number of primary clinical trials under the PIONEER and SUSTAIN programmes, meaning that we were limited in the overall design of our study—especially related to the recruitment of participants to each age subgroup. Due to the nature of this *post hoc* study, a longer follow‐up period for younger‐aged adults was not possible but would have been beneficial in assessing long‐term effects in the younger‐aged cohort. Whereas certain studies in the general literature use age at diagnosis for recruitment, the SUSTAIN and PIONEER programmes focused on current age. This approach has certain benefits, including our study presenting data on young adults that would not otherwise be represented in other clinical trials and that current age may be a more pragmatic representation of participants' current T2D status because both physiological and ageing‐related factors evolve with life circumstances and can interact with treatment responses [1, 57]. On the other hand, using this approach does not account for diabetes duration, which may also affect disease progression and outcomes, and age at diagnosis is a commonly used qualifier for subgrouping participants with T2D in other studies, making our results less comparable to other studies [58]. Because this study relied on data from these previously completed trials, it was not possible to recruit participants evenly to each of the age subgroups. As a result, the size of each subgroup varied, with a larger proportion of the participants belonging to the > 50 years subgroup, which led to wider confidence intervals and a reduced statistical power in the comparison between subgroups. However, this was partly mitigated by our analysis covering several studies, which provided largely consistent results across all the studies.

The greater reductions in HbA_1c_ observed in the younger age group may, to some extent, be related to treatment history and concomitant therapy, which may differ systematically by age subgroup. However, the clinical trials in this study included the same inclusion and exclusion criteria for concomitant medications, irrespective of age subgroup. This means that all adults were allowed (or disallowed) to use the same concomitant medications upon inclusion in the study. We did not perform any additional sub‐analyses based on concomitant medications because this would have further limited the statistical power in the comparison between subgroups. Additionally, we did not perform any further sub‐analyses by race/ethnicity for the same reason that additional analyses would further split the younger‐aged cohort into smaller subgroups that would prevent meaningful interpretation. Moreover, a *post hoc* analysis focusing on the impact of race/ethnicity has already been performed on the SUSTAIN trials, which found that semaglutide was associated with consistent and clinically relevant reductions in HbA_1c_ and BW in participants with T2D, with minor variations in efficacy and safety outcomes associated with race/ethnicity [59]. Inherent to the subgrouping of participants by age, the younger‐aged subgroup consists of a potentially heterogeneous cohort of adults aged 18–40 years, meaning that this age group could be heterogeneous in the spread of actual ages. However, we grouped adults into the younger‐aged subgroup to answer the question of how T2D management with semaglutide affects participants in this younger adult cohort that is severely underrepresented in clinical trials, and further subgrouping by actual age would have reduced statistical power and prevented meaningful interpretation.

There was a numerical imbalance noted in the incidence rate of SAEs in younger adults receiving s.c. semaglutide treatment compared to the comparator arm. However, this observation was not consistent in younger adults treated with oral semaglutide. The percentage of younger adults reporting SAEs was similar, and the numerical imbalance in incidence rates was driven by a few participants reporting several symptoms in the same case. Importantly, there was no overall imbalance in percentages for SAEs between arms and formulations, and no new safety concerns have been identified in the current analysis. For GI AEs leading to discontinuation, there was a smaller proportion of participants in the younger‐aged cohort who discontinued treatment across both programmes. However, the younger‐adult subgroup is proportionally smaller than the older age subgroups, and safety observations for subgroups should therefore be interpreted with caution, as the trials were not powered for these analyses.

Importantly, the overall imbalance in the number of participants in the younger‐aged subgroup compared to the other age subgroups reflects the underrepresentation of the younger‐aged cohort in T2D studies. This is also relevant for the PIONEER and SUSTAIN programmes, given that there were no specific exclusion criteria precluding their involvement. Comparatively, a recent post hoc analysis of the SURPASS trials has shown that the SURPASS programme has included approximately 20% participants with EOT2D [44]. However, the SURPASS programme used age of diabetes onset as a categorical marker, whereas our study used current age at the time of the trials. To this point, the average age in the EOT2D group for the SURPASS‐2 and ‐3 trials was > 40 years, which corresponds to our middle‐aged subgroup. Moreover, the current study expands the knowledge of age‐stratified T2D management with regards to GLP‐1 RA treatment, whereas the SURPASS programme addressed T2D management with respect to the use of a GIP/GLP‐1 dual agonist. Furthermore, the current study expands on the knowledge of oral formulations of GLP‐1 RA‐based treatments by highlighting the efficacy in younger adults with T2D. With regards to the low number of participants observed in the younger‐aged cohort, contributing factors may include the complex and busy lives and competing priorities for younger adults, which may make it challenging for them to participate in studies, as well as the lower perceived vulnerability to T2D at a younger age, and the fact that the age from 18 to 40 years represents the childbearing age for women, which often precludes involvement due to pregnancy being a typical exclusion criteria in clinical trials [4]. A further explanation may be due to the prevalence of T2D, which is more common in older age groups compared to younger adults [60], despite rapidly increasing rates in younger adults [1, 2, 3, 5].

Of note, there was a shorter duration of diabetes in the younger‐aged cohort. There is a positive association between the duration of diabetes and the deterioration of β‐cell function [11]. Furthermore, a younger age of T2D onset is associated with a prolonged, gradual decline in β‐cell function throughout life [6]. This is particularly relevant given that the glucose‐lowering effect of GLP‐1 RAs is known to be dependent on β‐cell functionality [61]. Younger individuals may therefore retain greater residual β‐cell function and metabolic responsiveness [61], which could contribute to a stronger glycaemic response to GLP‐1 RA therapy. Despite having similar baseline levels of HbA_1c_, there was a tendency for a greater reduction in HbA_1c_ observed in the younger‐aged cohort. Therefore, this greater reduction observed in the younger‐aged subgroup may partially be explained by the relatively shorter disease duration experienced by the younger‐aged cohort. However, further studies are required to determine whether the indication of increased efficacy observed in the younger‐aged subgroup is dependent on the duration of diabetes or due to a younger age of diagnosis.

In conclusion, younger adults living with T2D are at high risk of future health complications and require evidence‐based interventions to manage this risk. Here, we present evidence that semaglutide treatment is safe and efficacious in reducing HbA_1c_ and BW across all age groups with T2D, and there is an indication that there is a greater effect in adults aged ≤ 40 years with T2D. Importantly, semaglutide is one of the few medications with evidence of efficacy in this age group. Further studies are needed to determine the long‐term impact of semaglutide on people with EOT2D, particularly to establish efficacy in reducing the development of T2D‐associated complications.

## Funding

This study was sponsored by Novo Nordisk.

## Conflicts of Interest

F.Z. acted as a consultant/advisor for Servier, Menarini, Daiichi Sankyo, and Adelphi Real World. M.J.D. has acted as a consultant/advisor and speaker for Eli Lilly, Novo Nordisk, and Sanofi, has attended advisory boards for Abbvie, Amgen, AstraZeneca, Biomea Fusion, Carmot/Roche, Sanofi, Zealand Pharma, Regeneron, GSK, and EktaH, and as a speaker for AstraZeneca and Boehringer Ingelheim. She has received grants from AstraZeneca, Boehringer Ingelheim, and Novo Nordisk. P.P., L.B., and E.I. are employees and shareholders of Novo Nordisk. E.G.A. was an employee of Novo Nordisk at the time of drafting this manuscript. V.R.A. has institutional contracts with Amgen, Applied Therapeutics, AstraZenaca, Biommea, Boehringer Ingelheim, Corcept, Eli Lilly, Fractyl, Novo Nordisk, Pfizer, Recordati, Rhythm, and Servier. V.R.A. has also served as a consultant for Baim Institute for Clinical Research, Mediflix, Roche, and Sanofi.

## Supporting information


**Table S1:** Study designs. Abbreviations: α‐GI, α‐glucosidase inhibitor; ALT, alanine aminotransferase; BMI, body‐mass index; CKD‐EPI, Chronic Kidney Disease Epidemiology Collaboration; CoEQ, Control of Eating Questionnaire; CRP, C‐reactive protein; DPP‐4, dipeptidyl peptidase‐4; DTR‐QOL, Diabetes Therapy‐Related Quality of Life; DTSQ, diabetes treatment satisfaction questionnaire; eGFR, estimated glomerular filtration rate; FFA, free fatty acid; FPG, fasting plasma glucose; GLP‐1 RA, glucagon‐like peptide‐1 receptor agonist; HbA_1c_, glycated haemoglobin A1c; HDL, high‐density lipoprotein; HOMA‐IR, homeostatic model assessment of insulin resistance; HOMA‐B, homeostasis model assessment of β‐cell function; hs, high sensitivity; IU, international unit; IWQOL, impact of weight on quality of life; LDL, low‐density lipoprotein; MDRD, Modification of Diet in Renal Disease; MEN2, multiple endocrine neoplasia type 2; MI, myocardial infarction; MTC, medullary thyroid carcinoma; NYHA, New York Heart Association; OAD, oral antidiabetic drug; OD, once daily; OW, once weekly; PAI‐1, plasminogen activator inhibitor‐1; PGI‐C, patient global impression of change; PGI‐S, patient global impression of severity; p.o. per oral; PRO, patient‐reported outcome; s.c. subcutaneous; SF‐36, short‐form 36; SGLT‐2, sodium‐glucose cotransporter 2; SMPG, self‐monitored blood glucose; SU, sulfonylurea; T2D, type 2 diabetes; TEAE, treatment‐emergent adverse event; TIA, transient ischaemic attack; TZD, thiazolidinedione; VLDL, very‐low density lipoprotein.
**Table S2:** Further study design details. Abbreviations: α‐GI, α‐glucosidase inhibitor; DPP‐4, dipeptidyl peptidase‐4; eGFR, estimated glomerular filtration rate; exenatide ER, exenatide extended release; GLP‐1RA, glucagon‐like peptide‐1 receptor agonist; HF, heart failure; MEN2, multiple endocrine neoplasia type 2; MET, metformin; MTC, medullary thyroid carcinoma; N, number of participants randomised; NYHA, New York Heart Association; OAD, oral antidiabetic drug; OD, once daily; OW, once weekly; p.o. per oral; s.c. subcutaneous; SU, sulphonylurea; T2D, type 2 diabetes; TZD, thiazolidinedione.
**Table S3:** Summary of absolute change in HbA1c by Trial. Abbreviations: CI, confidence interval; IGlar, insulin glargine; MRCT, multi‐regional clinical trial; OAD, oral anti‐diabetic.
**Table S4:** Summary of HbA1c endpoints. Abbreviations: CI, confidence interval; MRCT, multi‐regional clinical trial; OAD, oral anti‐diabetic.
**Table S5:** Summary of absolute change in body weight by trial. Abbreviations: CI, confidence interval; IGlar, insulin glargine; MRCT, multi‐regional clinical trial; OAD, oral anti‐diabetic.
**Table S6:** Summary of Body Weight Endpoints. Abbreviations: CI, confidence interval; MRCT, multi‐regional clinical trial; OAD, oral anti‐diabetic.
**Table S7:** SAEs in SUSTAIN and PIONEER programmes. † indicates percentage of participants with events and the rate of events per 100 patient‐years of exposure, which were corrected using a Cochrane‐Mantel–Haenszel adjustment.
**Table S8:** GI SAEs in the SUSTAIN and PIONEER programmes. † indicates percentage of participants with events and the rate of events per 100 patient‐years of exposure, which were corrected using a Cochrane‐Mantel–Haenszel adjustment.
**Table S9:** GI AEs events leading to discontinuation in subcutaneously‐treated participants. † indicates percentage of participants with events and the rate of events per 100 patient‐years of exposure, which were corrected using a Cochrane‐Mantel–Haenszel adjustment. The analyses for gastrointestinal serious adverse events were performed using SAS and on‐treatment data. Abbreviations: AE, adverse event; GI, gastrointestinal.
**Table S10:** GI AEs events leading to discontinuation in orally‐treated participants.
^†^ indicates percentage of participants with events and the rate of events per 100 patient‐years of exposure, which were corrected using a Cochrane‐Mantel–Haenszel adjustment. The analyses for gastrointestinal serious adverse events were performed using SAS and on‐treatment data. Abbreviations: AE, adverse event; GI, gastrointestinal.

## Data Availability

An authorised researcher can request access to clinical study data by submitting a research proposal for review and approval by Novo Nordisk and an internal independent review panel. Requests are usually considered after the research is finished and the main results have been published. If the research supports a regulatory application, requests will be considered after the product and its intended use are approved in both the EU and the USA. Participants clinical data will be anonymised, following an approved internal process, before data are shared to external third parties. For details on how to request access to clinical data, visit novonordisk‐trials.com.
