# Supplementary material for: Semaglutide Treatment in Young Adults Living With Type 2 Diabetes: A Post Hoc Analysis From the SUSTAIN and PIONEER Clinical Trials
Source: Diabetes Obes Metab. 2026 Apr 17;28(7):5787–802. doi: 10.1111/dom.70770 (PMC13243996; doi:10.1111/dom.70770)
Supplement: Supplementary file 1 — Table S1: Study designs. Abbreviations: α‐GI, α‐glucosidase inhibitor; ALT, alanine aminotransferase; BMI, body‐mass index; CKD‐EPI, Chronic Kidney Disease Epidemiology Collaboration; CoEQ, Control of Eating Questionnaire; CRP, C‐reactive protein; DPP‐4, dipeptidyl peptidase‐4; DTR‐QOL, Diabetes Therapy‐Related Quality of Life; DTSQ, diabetes treatment satisfaction questionnaire; eGFR, estimated glomerular filtration rate; FFA, free fatty acid; FPG, fasting plasma glucose; GLP‐1 RA, glucagon‐like peptide‐1 receptor agonist; HbA1c, glycated haemoglobin A1c; HDL, high‐density lipoprotein; HOMA‐IR, homeostatic model assessment of insulin resistance; HOMA‐B, homeostasis model assessment of β‐cell function; hs, high sensitivity; IU, international unit; IWQOL, impact of weight on quality of life; LDL, low‐density lipoprotein; MDRD, Modification of Diet in Renal Disease; MEN2, multiple endocrine neoplasia type 2; MI, myocardial infarction; MTC, medullary thyroid carcinoma; NYHA, New York Heart Association; OAD, oral antidiabetic drug; OD, once daily; OW, once weekly; PAI‐1, plasminogen activator inhibitor‐1; PGI‐C, patient global impression of change; PGI‐S, patient global impression of severity; p.o. per oral; PRO, patient‐reported outcome; s.c. subcutaneous; SF‐36, short‐form 36; SGLT‐2, sodium‐glucose cotransporter 2; SMPG, self‐monitored blood glucose; SU, sulfonylurea; T2D, type 2 diabetes; TEAE, treatment‐emergent adverse event; TIA, transient ischaemic attack; TZD, thiazolidinedione; VLDL, very‐low density lipoprotein. Table S2: Further study design details. Abbreviations: α‐GI, α‐glucosidase inhibitor; DPP‐4, dipeptidyl peptidase‐4; eGFR, estimated glomerular filtration rate; exenatide ER, exenatide extended release; GLP‐1RA, glucagon‐like peptide‐1 receptor agonist; HF, heart failure; MEN2, multiple endocrine neoplasia type 2; MET, metformin; MTC, medullary thyroid carcinoma; N, number of participants randomised; NYHA, New York Heart Association; OAD, oral antidia [file DOM-28-5787-s001.docx]

# Semaglutide treatment in young adults living with type 2 diabetes: A Post-Hoc Analysis from the SUSTAIN and PIONEER Clinical Trials

### Supplementary Appendix

### Supplementary Table 1: Study Designs

| **Trial** | **Semaglutide Treatment** | **Comparator Treatment** | **Primary endpoint** | **Confirmatory secondary endpoints** | **Supportive secondary endpoints** | **Inclusion Criteria** | **Exclusion Criteria** |
| --- | --- | --- | --- | --- | --- | --- | --- |
| **SUSTAIN 1** | Semaglutide 0.5 mg OW s.c., Semaglutide 1.0 mg OW s.c. | Placebo | Change from baseline to week 30 in HbA_1c_ | Change from baseline to week 30 in body weight (kg) | Change from baseline to week 30 in:   - FPG - 7-point SMPG profile (Mean 7-point profile and mean postprandial increment over all meals) - Insulin, C-peptide, glucagon, pro-insulin, pro-insulin /insulin ratio, homeostasis model assessment of beta-cell function (HOMA-B) and insulin resistance (HOMA-IR) (all fasting) - Fasting blood lipids (total cholesterol, LDL-cholesterol, VLDL-cholesterol, HDL-cholesterol, triglycerides and FFA) - BMI and waist circumference - Systolic and diastolic blood pressure | - Male or female, aged ≥18 years at the time of signing inform consent - For Japan only: Male or female, aged ≥20 years at the time of signing inform consent - Subjects diagnosed with T2D and treated with diet and exercise for at least 30 days before screening - HbA_1c_ 7.0–10.0% (53-86 mmol /mol) (both inclusive) | - Any chronic disorder or severe disease which, in the opinion of the investigator, might jeopardise subject’s safety or compliance with the protocol - Treatment with any glucose lowering agent(s) in a period of 90 days prior to screening. An exception is short-term treatment (≤7 days in total) with insulin in connection with inter-current illness - History of chronic or idiopathic acute pancreatitis - Screening calcitonin value ≥50 ng/L (pg/mL) - Personal or family history of MTC or MEN 2 - Impaired renal function defined as eGFR <30 mL/min/1.73 m^2^ per MDRD formula (4 variable version) - Acute coronary or cerebrovascular event within 90 days before randomisation - Heart failure, NYHA class IV - Known proliferative retinopathy or maculopathy requiring acute treatment according to the opinion of the investigator - Diagnosis of malignant neoplasm in the previous 5 years (except basal cell skin cancer or squamous cell skin cancer) |
| **SUSTAIN 2** | Semaglutide 0.5 mg OW s.c. + placebo, Semaglutide 1.0 mg OW s.c. + placebo | Sitagliptin 100 mg p.o. OD + placebo | Change from baseline to week 56 in HbA_1c_ | Change from baseline to week 56 in body weight (kg) | Change from baseline to week 56 in:   - FPG - 7-point SMPG profile (Mean 7-point profile and mean postprandial increment over all meals) - Insulin, C-peptide, glucagon, pro-insulin, pro-insulin /insulin ratio, homeostasis model assessment of beta-cell function (HOMA-B) and insulin resistance (HOMA-IR) (all fasting) Fasting blood lipids (total cholesterol, LDL-cholesterol, VLDL-cholesterol, HDL-cholesterol, triglycerides and FFA) - BMI and waist circumference - Systolic and diastolic blood pressure - hs-CRP - PROs (SF-36v2™ and DTSQ scores) | - Male or female, aged ≥18 years at the time of signing informed consent - For Japan only: Aged ≥20 years - Subjects diagnosed with T2D and on stable treatment in a period of 90 days prior to screening with either metformin ≥1500 mg (or maximum tolerated dose), pioglitazone ≥30 mg (or maximum tolerated dose), rosiglitazone ≥4 mg (or maximum tolerated dose) or a combination of either metformin /pioglitazone or metformin /rosiglitazone (doses as for individual therapies). Stable is defined as unchanged medication and unchanged dose - HbA_1c_ 7.0–10.5% (53-91 mmol/mol) (both inclusive) | - Any chronic disorder or severe disease which, in the opinion of the investigator, might jeopardise subject’s safety or compliance with the protocol - Treatment with any glucose lowering agent(s) in a period of 90 days prior to screening. An exception is short-term treatment (≤7 days in total) with insulin in connection with inter-current illness - History of chronic or idiopathic acute pancreatitis - Screening calcitonin value ≥50 ng/L (pg/mL) - Personal or family history of MTC or MEN 2 - Impaired renal function defined as eGFR <30 mL/min/1.73 m^2^ per MDRD formula (4 variable version) - Acute coronary or cerebrovascular event within 90 days before randomisation - Heart failure, NYHA class IV - Known proliferative retinopathy or maculopathy requiring acute treatment according to the opinion of the investigator - Diagnosis of malignant neoplasm in the previous 5 years (except basal cell skin cancer or squamous cell skin cancer) |
| **SUSTAIN 3** | Semaglutide 1.0 mg OW s.c. | Exenatide ER 2.0 mg OW s.c. | Change from baseline to week 56 in HbA_1c_ | Change from baseline to week 56 in body weight (kg) | Change from baseline to week 56 in:   - FPG - 7-point SMPG profile (Mean 7-point profile and mean postprandial increment over all meals) - Insulin, C-peptide, glucagon, pro-insulin, pro-insulin /insulin ratio, homeostasis model assessment of beta-cell function (HOMA-B) and insulin resistance (HOMA-IR) (all fasting) - Fasting blood lipids (total cholesterol, LDL-cholesterol, VLDL-cholesterol, HDL-cholesterol, triglycerides and FFA) - BMI and waist circumference - Systolic and diastolic blood pressure - hs-CRP - PROs (SF-36v2™ and DTSQ scores) | - Male or female, aged ≥18 years at the time of signing informed consent - Subjects diagnosed with T2D and on stable diabetes treatment with 1-2 OADs (Metformin ≥1500 mg or maximum tolerated dose and /or TZD and SUs ≥ half of maximum dose allowed according to national label) for at least 90 days prior to screening. Stable is defined as unchanged medication and unchanged dose - HbA_1c_ 7.0–10.5% (53–91 mmol/mol) (both inclusive) | - Any chronic disorder or severe disease which, in the opinion of the investigator, might jeopardise subject’s safety or compliance with the protocol - Treatment with glucose lowering agent(s) other than stated in the inclusion criteria in a period of 90 days before screening. An exception is short-term treatment (≤7 days in total) with insulin in connection with inter-current illness - History of chronic or idiopathic acute pancreatitis - Screening calcitonin value ≥50 ng/L (pg/mL) - Personal or family history of MTC or MEN 2 - Impaired renal function defined as eGFR <60 mL/min /1.73 m^2^ per MDRD formula (4 variable version) - Acute coronary or cerebrovascular event within 90 days before randomisation - Heart failure, NYHA class IV - Known proliferative retinopathy or maculopathy requiring acute treatment according to the opinion of the investigator - Diagnosis of malignant neoplasm in the previous 5 years (except basal cell skin cancer or squamous cell skin cancer) |
| **SUSTAIN 4** | Semaglutide 0.5 mg OW s.c., Semaglutide 1.0 mg OW s.c. | Insulin glargine starting from 10 IU OD | Change from baseline to week 30 in HbA_1c_ | Change from baseline to week 30 in body weight (kg) | Change from baseline to week 30 in:   - FPG - 8-point SMPG profile (Mean 8-point profile and mean postprandial increment over all meals) - Fasting blood lipids (total cholesterol, LDL-cholesterol, VLDL-cholesterol, HDL-cholesterol, triglycerides, FFA) - BMI and waist circumference - hs-CRP, PAI-1 - Systolic and diastolic blood pressure - PROs (SF-36v2™ and DTSQ scores) | - Male or female, aged ≥18 years at the time of signing informed consent - Insulin-naïve subjects diagnosed withT2D and on stable diabetes treatment with metformin or metformin and SU (metformin ≥1500 mg or maximum tolerated dose and SU ≥ half of maximum allowed dose according to national label) for at least 90 days before screening. Stable is defined as unchanged medication and unchanged dose - HbA_1c_ 7.0–10.0% (53-86 mmol/mol) both inclusive | - Any chronic disorder or severe disease which, in the opinion of the investigator, might jeopardise subject’s safety or compliance with the protocol - Treatment with any glucose lowering agent(s) other than stated in the inclusion criteria in a period of 90 days before screening. An exception is short-term treatment (≤7 days in total) with insulin in connection with intercurrent illness - Experienced more than 3 episodes of severe hypoglycaemia within 6 months prior to screening, and/or hypoglycaemia unawareness - History of chronic or idiopathic acute pancreatitis - Screening calcitonin value ≥50 ng/L - Personal or family history of MTC or MEN2 - Severe renal impairment defined as eGFR <30 mL/min/1.73 m^2^ per MDRD formula (4 variable version) - Acute coronary or cerebrovascular event within 90 days before randomisation - Heart failure, New York Heart Association Class IV - Known proliferative retinopathy or maculopathy requiring acute treatment according to the opinion of the investigator - Diagnosis of malignant neoplasm in the previous 5 years (except basal cell skin cancer or squamous cell skin cancer) |
| **SUSTAIN 5** | Semaglutide 0.5 mg OW s.c., Semaglutide 1.0 mg OW s.c. | Placebo | Change from baseline to week 30 in HbA_1c_ | Change from baseline to week 30 in body weight (kg) | Change from baseline to week 30 in:   - FPG - Insulin dose - 7-point SMPG profile (Mean 7-point profile and mean postprandial increment over all meals) - Fasting blood lipids (total cholesterol, LDL cholesterol, VLDL cholesterol, HDL cholesterol, triglycerides, FFA) - BMI and waist circumference - hs-CRP - Systolic and diastolic blood pressure - PROs (SF-36v2™ and DTSQ scores) | - Male or female, aged ≥18 years at the time of signing inform consent. For Japan: Male or female, aged ≥20 years at the time of signing informed consent - Subjects diagnosed with T2D and on stable diabetes treatment (+/- 20% change in total daily dose) with basal insulin (minimum of 0.25 IU/kg/day and/or 20 IU/day of: insulin glargine, insulin detemir, insulin degludec and/or NPH insulin) alone or in combination with metformin (minimum of 1500 mg/day or maximal tolerable dose) for 90 days prior to screening - HbA_1c_ 7.0–10.0% (53-86 mmol/mol) both inclusive | - Any chronic disorder or severe disease which, in the opinion of the investigator, might jeopardise subject’s safety or compliance with the protocol - Treatment with any glucose lowering agents other than stated in the inclusion criteria in a period of 90 days before screening. An exception is short-term treatment (≤7 days in total) with bolus insulin in connection with intercurrent illness - Experienced more than 3 episodes of severe hypoglycaemia within 6 months prior to screening, and/or hypoglycaemia unawareness - History of pancreatitis (acute or chronic) - Screening calcitonin value ≥50 ng/L (pg/mL) - Personal or family history of MTC or MEN 2 - Severe renal impairment defined as eGFR <30 mL/min/1.73 m^2^ per MDRD (4 variable version) - Acute coronary or cerebrovascular event within 90 days before randomisation - Heart failure, NYHA Class IV - Known proliferative retinopathy or maculopathy requiring acute treatment according to the opinion of the investigator - Diagnosis of malignant neoplasm in the previous 5 years (except basal cell skin cancer or squamous cell skin cancer) |
| **SUSTAIN 7** | Semaglutide 0.5 mg OW s.c., Semaglutide 1.0 mg OW s.c. | Dulaglutide 0.75 mg OW s.c., Dulaglutide 1.5 mg OW s.c. | Change from baseline to week 40 in HbA_1c_ | Change from baseline to week 40 in body weight (kg) | Change from baseline to week 40 in:   - FPG - 7-point SMPG profile (Mean 7-point profile and mean postprandial increment over all meals) - Fasting blood lipids (total cholesterol, LDL cholesterol, HDL cholesterol, triglycerides) - BMI and waist circumference - Systolic and diastolic blood pressure - PROs (SF-36v2™ and DTSQ) | - Male or female, aged ≥18 years at the time of signing informed consent - Subjects with T2D diagnosed clinically ≥90 days prior to screening - HbA_1c_ 7.0–10.5% (53–91 mmol/mol) (both inclusive) - Subjects on stable diabetes treatment with metformin (minimum of 1500 mg/day or maximal tolerated dose documented in the patient medical record) for 90 days prior to screening | - Treatment with any medication for the indication of diabetes or obesity other than stated in the inclusion criteria in a period of 90 days before screening. An exception is short-term insulin treatment for acute illness for a total of ≤14 days - History of pancreatitis (acute or chronic) - Screening calcitonin ≥50 ng/L - Family or personal history of MTC or MEN 2. - Renal impairment defined as eGFR <60 mL/min/1.73 m^2^ as per CKD-EPI - Any of the following: MI, stroke or hospitalisation for unstable angina and/or TIA within the past 180 days prior to the day of screening - Subjects presently classified as being in NYHA Class IV - Planned coronary, carotid or peripheral artery revascularisation on the day of screening - Proliferative retinopathy or maculopathy requiring acute treatment - History or presence of malignant neoplasms within the last 5 years (except basal and squamous cell skin cancer and *in-situ* carcinomas) |
| **SUSTAIN 9** | Semaglutide 1.0 mg OW s.c. | Placebo | Change from baseline to week 30 in HbA_1c_ | Change from baseline to week 30 in body weight (kg) | Change from baseline to week 30 in:   - FPG - 7-point SMPG profile (Mean 7-point profile and mean postprandial increment over all meals) - Fasting blood lipids (total cholesterol, LDL cholesterol, HDL cholesterol, triglycerides) - Body weight (%) - BMI and waist circumference - Systolic and diastolic blood pressure - PROs (SF-36v2^TM^ and DTSQ) | - Male or female, aged ≥18 years at the time of signing informed consent. For Japan only: Male or female, age ≥ 20 years at the time of signing informed consent - Diagnosed with T2D - HbA_1c_ of 7.0-10.0% (53-86 mmol/mol) (both inclusive) - Stable dose of an SGLT-2 inhibitor as monotherapy or in combination (including fixed-dose drug combination) with a stable dose of metformin (≥ 1500 mg or maximum tolerated dose) or a SU for at least 90 days prior to the day of screening. All medications in compliance with current local label | - Any chronic disorder or severe disease which, in the opinion of the investigator, might jeopardise subject’s safety or compliance with the protocol - ALT >2.5x UNL - Family or personal history of MEN 2 or MTC. Family is defined as a first degree relative - History or presence of pancreatitis (acute or chronic) - History of diabetic ketoacidosis - Any of the following: MI, stroke, hospitalization for unstable angina or TIA within the past 180 days prior to the day of screening - Subjects presently classified as being in NYHA Class IV - Planned coronary, carotid or peripheral artery revascularisation known on the day of screening - Renal impairment measured as eGFR value < 60 ml/min/1.73 m^2^ as defined by KDIGO 2012 classification using isotope dilution mass spectrometry for serum creatinine measured at screening - Treatment with any medication for the indication of diabetes or obesity other than stated in the inclusion criteria within the past 90 days prior to the day of screening. However, short term insulin treatment for a maximum of 14 days prior to the day of screening is allowed - Proliferative retinopathy or maculopathy requiring acute treatment. Verified by fundus photography or dilated fundoscopy performed within the past 90 days prior to randomisation - Presence or history of malignant neoplasms within the past 5 years prior to the day of screening. Basal and squamous cell skin cancer and any carcinoma *in-situ* is allowed |
| **SUSTAIN 10** | Semaglutide 1.0 mg OW s.c. | Liraglutide 1.2 mg s.c. OD | Change from baseline to week 30 in HbA_1c_ | Change from baseline to week 30 in body weight (kg) | Change from baseline to week 30 in:   - FPG - 7-point SMPG profile (Mean 7-point profile and mean postprandial increment over all meals - Fasting blood lipids (total cholesterol, LDL-cholesterol, HDL-cholesterol, triglycerides) - BMI and waist circumference - Systolic and diastolic blood pressure - Body weight (%) - PROs (SF-36v2^TM^ and DTSQ) | - Male or female, aged ≥18 years at the time of signing informed consent - Diagnosed with T2D - HbA_1c_ of 7.0-11.0% (53−97 mmol/mol) (both inclusive) - Stable daily dose(s) including any of the following anti-diabetic drug(s) or combination regimens 90 days prior to the day of screening: a) Biguanides (metformin ≥1500 mg or maximum tolerated dose documented in the subject's medical record ), b) SU (≥ half of the maximum approved dose according to local label or maximum tolerated dose as documented in subject medical record), or c) SGLT-2 inhibitors (≥ half of the maximum approved dose according to local label or maximum tolerated dose as documented in subject medical record) | - Any condition, which in the investigator’s opinion might jeopardise subject’s safety or compliance with the protocol - Family or personal history of MEN 2 or MTC. Family is defined as a first degree relative - History or presence of pancreatitis (acute or chronic) - History of diabetic ketoacidosis - Any of the following: MI, stroke, hospitalization for unstable angina or TIA within the past 180 days prior to the day of screening - Subjects presently classified as being in NYHA Class IV - Planned coronary, carotid or peripheral artery revascularisation known on the day of screening - Renal impairment measured as eGFR value <30 ml/min/1.73 m^2^ as defined by KDIGO 2012 classification - Impaired liver function, defined as ALT ≥2.5 x UNL at screening - Treatment with any medication for the indication of diabetes or obesity other than stated in the inclusion criteria within the past 90 days prior to the day of screening. However, short term insulin treatment for a maximum of 14 days and previous insulin treatment for gestational diabetes prior to the day of screening is allowed - Proliferative retinopathy or maculopathy requiring acute treatment. Verified by fundus photography or dilated fundoscopy performed within the past 90 days prior to randomisation - Presence or history of malignant neoplasms within the past 5 years prior to the day of screening. Basal and squamous cell skin cancer and any carcinoma *in-situ* is allowed |
| **SUSTAIN Japan OAD combination** | Semaglutide 0.5 mg OW s.c., Semaglutide 1.0 mg OW s.c. | Additional OAD (α-GI, MET, DPP-4 inhibitor, glinide, SU, or TZD) | Number of TEAEs during 56 weeks of treatment |  | Change from baseline to week 56 in:   - HbA_1c_ - Body weight (kg) - FPG - 7-point SMPG profile (Mean 7-point profile and mean postprandial increment over all meals) - Insulin, C-peptide, glucagon, pro-insulin, pro-insulin /insulin ratio, homeostasis model assessment of beta-cell function (HOMA-B) and insulin resistance (HOMA- IR) (all fasting) - Fasting blood lipids (total cholesterol, LDL-cholesterol, VLDL-cholesterol, HDL-cholesterol, triglyceride, and FFA) - BMI and waist circumference - Systolic and diastolic blood pressure - hs-CRP | - Male or female, aged ≥ 20 years at the time of signing informed consent - HbA_1c_ between 7.0% and 10.5% (53–91 mmol/mol) (both inclusive) - Japanese subjects with T2D (diagnosed clinically) and on stable treatment (either a) on diet and exercise therapy for at least 30 days before Visit 1 (week -2) or b) on OAD monotherapy (either of SU, glinide, α-GI or TZD) within approved Japanese labelling in addition to diet and exercise therapy for at least 60 days before Visit 1 (week -2) | - Any disorder which, in the opinion of the investigator, might jeopardise subject’s safety or compliance with the protocol - Treatment with glucose lowering agent(s) other than stated in the inclusion criteria within 60 days before Visit 1 (week −2) and treatment with once weekly GLP-1 RAs within 90 days before Visit 1 (week −2). An exception is short-term treatment (≤7 days in total) with insulin in connection with inter-current illness - History of chronic or idiopathic acute pancreatitis - Screening calcitonin value ≥50 ng/L (pg/mL) - Personal or family history of MTC or MEN2 - Impaired renal function defined as eGFR <30 mL/min/1.73 m^2^ per modification of diet in renal disease formula (4 variable version) - Acute coronary or cerebrovascular event within 90 days before randomisation (Visit 2 [week 0]) - Heart failure, NYHA class IV - Known proliferative retinopathy or maculopathy requiring acute treatment according to the opinion of the investigator - Diagnosis of malignant neoplasm in the previous 5 years (except basal cell skin cancer or squamous cell skin cancer) |
| **SUSTAIN Japan Monotherapy** | Semaglutide 0.5 mg OW s.c., Semaglutide 1.0 mg OW s.c. | Sitagliptin 100 mg p.o. OD | Number of TEAEs during 30 weeks of treatment |  | Change from baseline to week 30 in:   - HbA_1c_ - Body weight (kg) - FPG - 7-point SMPG profile (Mean 7-point profile and mean postprandial increment over all meals) - Fasting blood lipids (total cholesterol, LDL-cholesterol, VLDL-cholesterol, HDL-cholesterol, triglyceride, and FFA) - BMI and waist circumference - Systolic and diastolic blood pressure - hs-CRP | - Male or female, aged ≥20 years at the time of signing informed consent - HbA_1c_ between 6.5% and 9.5% (48–80 mmol/mol) (both inclusive) for subjects treated with OAD monotherapy and between 7.0% and 10.5% (53–91 mmol/mol) (both inclusive) for subjects treated with diet and exercise therapy at screening - Japanese subjects diagnosed with T2D who are: a) on stable OAD monotherapy at a half-maximum dose or below according to the approved Japanese labelling in addition to diet and exercise therapy for at least 30 days prior to screening (week −8) (For metformin only: the maximum dose of 750 mg /day is allowed except for METGLUCO®. For METGLUCO®, the allowable half-max dose of 1125 mg /day must be applied). ‘Stable’ is defined as unchanged medication and unchanged dose. or b) on stable diet and exercise therapy for at least 30 days prior to screening (week −2) | - Any disorder which, in the opinion of the investigator, might jeopardise the subject's safety or compliance with the protocol - Treatment with once-weekly GLP-1 RAs within 90 days prior to screening - Treatment with any glucose lowering agent(s) (except for pre-trial OAD for subject treated with OAD monotherapy) in a period of 60 days prior to screening. An exception is short-term treatment (≤7 days in total) with insulin in connection with inter-current illness - History of chronic or idiopathic acute pancreatitis - Screening calcitonin value ≥50 ng/L (pg/mL) - Personal or family history of MTC or MEN2 - Impaired renal function defined as eGFR <60 ml/min/1.73 m^2^ per modification of diet in renal disease formula (4 variable version) - Acute coronary or cerebrovascular event within 90 days before randomisation - Heart failure, NYHA class IV - Known proliferative retinopathy or maculopathy requiring acute treatment according to the opinion of the investigator - Diagnosis of malignant neoplasm in the previous 5 years (except basal cell skin cancer or squamous cell skin cancer) |
| **SUSTAIN – China MRCT** | Semaglutide 0.5 mg OW s.c. + placebo, Semaglutide 1.0 mg OW s.c. + placebo | Sitagliptin p.o. OD + placebo | Change from baseline to week 30 in HbA_1c_ | Change from baseline to week 30 in body weight (kg) | Change from baseline to week 30 in:   - FPG - 7-point SMPG profile (Mean 7-point profile and mean postprandial increment over all meals) - Insulin, C-peptide, glucagon, pro-insulin, pro-insulin/insulin ratio, homeostasis model assessment of beta-cell function (HOMA-B) and insulin resistance (HOMA-IR) (all fasting) - Fasting blood lipids (total cholesterol, LDL cholesterol, VLDL cholesterol, HDL cholesterol, triglycerides and FFA) - BMI and waist circumference - Systolic and diastolic blood pressure - hs-CRP - PROs (SF-36v2™ score, and DTSQ score) | - Male or female, aged ≥18 years at the time of signing informed consent - Subjects diagnosed withT2D and on stable treatment in a period of 60 days prior to screening with metformin ≥1500 mg (or maximum tolerated dose ≥1000 mg). Stable is defined as unchanged medication and unchanged daily dose - HbA_1c_ 7.0–10.5% (53-91 mmol/mol) (both inclusive) | - Any disorder which, in the opinion of the investigator, might jeopardise subject’s safety or compliance with the protocol - Treatment with glucose lowering agent(s) other than stated in the inclusion criteria in a period of 60 days before screening. An exception is short-term treatment (≤7 days in total) with insulin in connection with inter-current illness - Use of non-herbal Chinese medicine or other non-herbal local medicine with unknown/unspecified content. Herbal traditional Chinese medicine or other local herbal medicines may, at the Investigator’s discretion, be continued throughout the trial - History of pancreatitis (acute or chronic) - Screening calcitonin value ≥50 ng/L (pg/mL) - Personal or family history of MTC or MEN 2 - Impaired renal function defined as eGFR <60 mL/min/1.73 m^2^ per modification of diet in renal disease formula (4 variable version) - Acute coronary or cerebrovascular event within 90 days before randomisation - Heart failure, NYHA class IV - Known proliferative retinopathy or maculopathy requiring acute treatment according to the opinion of the investigator - Diagnosis of malignant neoplasm in the previous 5 years (except basal cell skin cancer or squamous cell skin cancer) |
| **PIONEER 1** | Semaglutide 3 mg p.o. OD, Semaglutide 7 mg p.o. OD, Semaglutide 14 mg p.o. OD | Placebo | Change from baseline to week 26 in HbA_1c_ | Change from baseline to week 26 in body weight (kg) | Change from baseline to week 26 in:   - FPG - Fasting C-peptide - Fasting insulin and proinsulin - Fasting glucagon - Insulin resistance (HOMA-IR) and beta-cell function (HOMA-B) - CRP - 7-point SMPG profile (Mean 7-point profile and mean postprandial increment over all meals) - Body weight (%) - BMI and waist circumference - Fasting lipid profile (total cholesterol, LDL cholesterol, HDL cholesterol and triglycerides) - PROs (SF-36v2^TM^, IWQOL-Lite, PGI-S items, and PGI-C items) | - Male or female, aged ≥18 years at the time of signing informed consent. For Japan only: Male or female, aged ≥20 years at the time of signing informed consent. For Algeria only: Male or female, aged ≥19 years at the time of signing informed consent - Diagnosed with T2D ≥30 days prior to day of screening - HbA_1c_ between 7.0-9.5% (53-80 mmol/mol) (both inclusive) - Treatment with diet and exercise for ≥30 days prior to day of screening | - Any disorder which, in the investigator’s opinion, might jeopardise subject’s safety or compliance with the protocol - Family or personal history of MEN 2 or MTC - History of pancreatitis (acute or chronic) - History of major surgical procedures involving the stomach potentially affecting absorption of trial product (e.g. subtotal and total gastrectomy, sleeve gastrectomy, gastric bypass surgery) - Any of the following: MI, stroke or hospitalisation for unstable angina or TIA within the past 180 days prior to the day of screening and randomisation - Subjects presently classified as being in NYHA Class IV - Planned coronary, carotid or peripheral artery revascularisation known on the day of screening - Subjects with ALT >2.5 x UNL - Renal impairment defined as eGFR <60 mL/min/1.73 m^2^ as per CKD-EPI formula - Treatment with any medication for the indication of diabetes or obesity in a period of 90 days before the day of screening. An exception is short-term insulin treatment for acute illness for a total of ≤14 days - Proliferative retinopathy or maculopathy requiring acute treatment. Verified by fundus photography or dilated fundoscopy performed within 90 days prior to randomisation - History or presence of malignant neoplasms within the last 5 years (except basal and squamous cell skin cancer and *in-situ* carcinomas) |
| **PIONEER 2** | Semaglutide 14 mg p.o. OD | Empagliflozin 25 mg p.o. OD | Change from baseline to week 26 in HbA_1c_ | Change from baseline to week 26 in body weight (kg) | Change from baseline to week 52 in:   - HbA_1c_ - Body weight (kg)   Change from baseline to week 26 and week 52 in:   - FPG - Fasting C-peptide - Fasting insulin and proinsulin - Fasting glucagon - Insulin resistance (HOMA-IR) and beta-cell function (HOMA-B) - 7-point SMPG profile (Mean 7-point profile and mean postprandial increment over all meals) - Body weight (%) - BMI and waist circumference - Fasting lipid profile (total cholesterol, LDL-cholesterol, VLDL-cholesterol, HDL-cholesterol, triglycerides, FFA) - CRP - PRO (SF-36v2^TM^ and CoEQ) | - Male or female, aged ≥18 years at the time of signing informed consent - Diagnosed with T2D ≥90 days prior to day of screening - HbA_1c_ of 7.0-10.5% (53-91 mmol/mol) (both inclusive) - Stable daily dose of metformin (≥1500 mg or maximum tolerated dose as documented in the subject medical record) ≥90 days prior to the day of screening | - Any disorder which, in the investigator’s opinion might jeopardise subject’s safety or compliance with the protocol - Family or personal history of MEN 2 or MTC - History of pancreatitis (acute or chronic) - History of major surgical procedures involving the stomach potentially affecting absorption of trial product (e.g. subtotal and total gastrectomy, sleeve gastrectomy, gastric bypass surgery) - Any of the following: MI, stroke or hospitalisation for unstable angina or TIA within the past 180 days prior to the day of screening - Subjects presently classified as being in NYHA Class IV - Planned coronary, carotid or peripheral artery revascularisation known on the day of screening - Subjects with ALT >2.5 x UNL - Renal impairment defined as eGFR <60 mL/min/1.73 m^2^ as per CKD-EPI formula - Treatment with any medication for the indication of diabetes or obesity other than stated in the inclusion criteria in a period of 90 days before the day of screening. An exception is short-term insulin treatment for acute illness for a total of ≤14 days - Proliferative retinopathy or maculopathy requiring acute treatment. Verified by fundus photography or dilated fundoscopy performed within 90 days prior to randomisation - History or presence of malignant neoplasms within the last 5 years (except basal and squamous cell skin cancer and carcinoma *in situ*) - History of diabetic ketoacidosis |
| **PIONEER 4** | Semaglutide 14 mg p.o. OD | Liraglutide 1.8 mg s.c. OD,  Placebo | Change from baseline to week 26 in HbA_1c_ | Change from baseline to week 26 in body weight (kg) | Change from baseline to week 52 in:   - HbA_1c_ - Body weight (kg)   Change from baseline to week 26 and week 52 in:   - FPG - 7-point SMPG profile (Mean 7-point profile and mean postprandial increment over all meals - Body weight (%) - BMI and waist circumference - Fasting lipid profile (total cholesterol, LDL-cholesterol, VLDL-cholesterol, HDL-cholesterol, triglycerides, FFA) - PRO (DTSQ scores) | - Male or female, aged ≥18 years at the time of signing informed consent. For Japan only: Male or female, age ≥20 years at the time of signing informed consent - Diagnosed with T2D ≥90 days prior to day of screening - HbA_1c_ of 7.0–9.5% (53-80.3 mmol/mol) (both inclusive) - Stable daily dose of metformin (≥1500 mg or maximum tolerated dose as documented in the subject medical record) alone or in combination with a stable daily dose of a SGLT-2 inhibitor (all doses approved as maintenance therapy) ≥90 days prior to the day of screening | - Any disorder which, in the investigator’s opinion, might jeopardise subject’s safety or compliance with the protocol - Family or personal history of MEN 2 or MTC - History of pancreatitis (acute or chronic) - History of major surgical procedures involving the stomach and potentially affecting absorption of trial product (e.g. subtotal and total gastrectomy, sleeve gastrectomy, gastric bypass surgery) - Any of the following: MI, stroke or hospitalisation for unstable angina or transient ischaemic attack within the past 180 days prior to the day of screening - Subjects presently classified as being in NYHA Class IV - Planned coronary, carotid or peripheral artery revascularisation known on the day of screening - Subjects with ALT >2.5 × UNL - Renal impairment defined as eGFR <60 mL/min /1.73 m^2^ as per CKD-EPI formula - Treatment with any medication for the indication of diabetes or obesity other than stated in the inclusion criteria in a period of 90 days before the day of screening. An exception is short-term insulin treatment for acute illness for a total of ≤14 days - Proliferative retinopathy or maculopathy requiring acute treatment. Verified by fundus photography or dilated fundoscopy performed within 90 days prior to randomisation - History or presence of malignant neoplasms within the last 5 years (except basal and squamous cell skin cancer and carcinoma *in situ*) - History of diabetic ketoacidosis |
| **PIONEER 7** | Semaglutide flexible dose (3, 7, or 14 mg) p.o. OD | Sitagliptin 100 mg p.o. OD | If a subject after week 52 achieves (yes /no) HbA_1c_ <7% (53 mmol/mol) (American Diabetes Association target). | Change from baseline to week 52 in body weight (kg) | Change from baseline to week 52 in:   - HbA_1c_ - FPG - Body weight (%) - BMI and waist circumference - Fasting lipid profile (total cholesterol, LDL-cholesterol, high-density, HDL-cholesterol, triglycerides). - PROs (SF-36v2^TM^ and DTSQ scores) | - Male or female, aged ≥18 years at the time of signing informed consent. For Korea only: Male or female, aged ≥19 years at the time of signing informed consent - Diagnosed with T2D ≥90 days prior to day of screening - HbA_1c_ 7.5-9.5% (58-80 mmol/mol) (both inclusive). - Treatment target of HbA_1c_ <7.0% (53 mmol/mol), as judged by the investigator - Stable daily dose(s) of 1-2 of the following anti-diabetic drugs within 90 days prior to the day of screening: a) Metformin (≥1500 mg or maximum tolerated dose documented in the subject medical record), b) SUs (≥ half of the maximum approved dose according to local label or maximum tolerated dose as documented in subject medical record), c) SGLT-2 inhibitors, or d) TZDs (≥ half of the maximum approved dose according to local label or maximum tolerated dose as documented in subject medical record) | - Any disorder which, in the investigator’s opinion, might jeopardise subject’s safety or compliance with the protocol - Family or personal history of MEN 2 or MTC - History of pancreatitis (acute or chronic) - History of major surgical procedures involving the stomach potentially affecting absorption of trial product (e.g. subtotal and total gastrectomy, sleeve gastrectomy, gastric bypass surgery) - Any of the following: MI, stroke or hospitalisation for unstable angina or TIA within the past 180 days prior to the day of screening and randomisation - Subjects presently classified as being in NYHA Class IV - Planned coronary, carotid or peripheral artery revascularisation known on the day of screening - Subjects with ALT >2.5 x UNL - Renal impairment defined as eGFR <60 mL/min/1.73 m^2^ as per CKD-EPI formula - Treatment with any medication for the indication of diabetes or obesity other than stated in the inclusion criteria in a period of 90 days before the day of screening. An exception is short-term insulin treatment for acute illness for a total of ≤14 days - Proliferative retinopathy or maculopathy requiring acute treatment. Verified by fundus photography or dilated fundoscopy performed within 90 days prior to randomisation - History or presence of malignant neoplasms within the last 5 years (except basal and squamous cell skin cancer and carcinoma *in situ*) - History of diabetic ketoacidosis |
| **PIONEER 8** | Semaglutide 3 mg p.o. OD, Semaglutide 7 mg p.o. OD, Semaglutide 14 mg p.o. OD | Placebo | Change from baseline to week 26 in HbA_1c_ | Change from baseline to week 26 in body weight (kg) | Change from baseline to week 52 in:   - HbA_1c_ - Body weight (kg)   Change from baseline to week 26 and 52 in:   - FPG - 7-point SMPG profile (Mean 7-point profile and mean postprandial increment over all meals) - Body weight (%) - BMI and waist circumference - Fasting lipid profile (total cholesterol, LDL cholesterol, HDL cholesterol, triglycerides, VLDL, FFA) - PROs (SF-36v2^TM^ health survey, IWQOL-Lite and DTSQ questionnaire scores) | - Male or female, aged ≥18 years at the time of signing informed consent. For Japan only: Male or female, aged ≥20 years at the time of signing informed consent - Diagnosed with T2D ≥90 days prior to the day of screening - HbA_1c_ of 7.0-9.5% (53-80 mmol/mol) (both inclusive) - Stable treatment with one of the following insulin regimens (minimum 10 IU/day) ≥90 days prior to the day of screening. Maximum 20% change in total daily dose is acceptable: basal insulin alone, basal and bolus insulin in any combination, or premixed insulin including combinations of soluble insulins. Concomitant treatment with stable daily dose of metformin (≥ 1500 mg or maximum tolerated dose as documented in the subject medical record) ≥ 90 days prior to the day of screening is allowed. For Japan only: Concomitant treatment with metformin is only allowed in combination with basal insulin alone (not in combination with basal-bolus or premixed insulin including combinations of soluble insulins) | - Any disorder which, in the investigator’s opinion, might jeopardise subject’s safety or compliance with the protocol - Family or personal history of MEN 2 or MTC - History of pancreatitis (acute or chronic) - History of major surgical procedures involving the stomach and potentially affecting absorption of trial product (e.g. subtotal and total gastrectomy, sleeve gastrectomy, gastric bypass surgery) - Any of the following: MI, stroke or hospitalisation for unstable angina or TIA within the past 180 days prior to the day of screening and randomisation - Classified as being in NYHA Class IV - Planned coronary, carotid or peripheral artery revascularisation known on the day of screening - Renal impairment defined as eGFR <60 mL/min/1.73 m^2^ as per CKD-EPI formula - Treatment with any medication for the indication of diabetes or obesity other than stated in the inclusion criteria in a period of 90 days before the day of screening. An exception is short-term change of insulin treatment for acute illness for a total of ≤14 days - Known hypoglycaemic unawareness according to Clarke’s questionnaire - Proliferative retinopathy or maculopathy requiring acute treatment. Verified by fundus photography or dilated fundoscopy performed within 90 days prior to randomisation - History or presence of malignant neoplasms within the last 5 years (except basal and squamous cell skin cancer and carcinoma *in situ*) - Subjects with ALT >2.5 x UNL |

Abbreviations: α-GI, α-glucosidase inhibitor; ALT, alanine aminotransferase; BMI, body-mass index; CKD-EPI, Chronic Kidney Disease Epidemiology Collaboration; CoEQ, Control of Eating Questionnaire; CRP, C-reactive protein; DPP-4, dipeptidyl peptidase-4; DTR-QOL, Diabetes Therapy-Related Quality of Life; DTSQ, diabetes treatment satisfaction questionnaire; eGFR, estimated glomerular filtration rate; FFA, free fatty acid; FPG, fasting plasma glucose; GLP-1 RA, glucagon-like peptide-1 receptor agonist; HbA_1c_, glycated haemoglobin A1c; HDL, high-density lipoprotein; HOMA-IR, homeostatic model assessment of insulin resistance; HOMA-B, homeostasis model assessment of β-cell function; hs, high sensitivity; IU, international unit; IWQOL, impact of weight on quality of life; LDL, low-density lipoprotein; MDRD, Modification of Diet in Renal Disease; MEN2, multiple endocrine neoplasia type 2; MI, myocardial infarction; MTC, medullary thyroid carcinoma; NYHA, New York Heart Association; OAD, oral antidiabetic drug; OD, once daily; OW, once weekly; PAI-1, plasminogen activator inhibitor-1; PGI-C, patient global impression of change; PGI-S, patient global impression of severity; p.o. per oral; PRO, patient-reported outcome; s.c. subcutaneous; SF-36, short-form 36; SGLT-2, sodium-glucose cotransporter 2; SMPG, self-monitored blood glucose; SU, sulfonylurea; T2D, type 2 diabetes; TEAE, treatment-emergent adverse event; TIA, transient ischaemic attack; TZD, thiazolidinedione; VLDL, very-low density lipoprotein.

### Supplementary Table 2: Further Study Design Details

| **Trial** | **Clinical Trial ID** | **Trial design** | **Background medication** | **N randomised** | **Semaglutide treatment** | **Comparator treatment** | **Treatment duration** |
| --- | --- | --- | --- | --- | --- | --- | --- |
| **SUSTAIN 1** | NCT02054897 | Double-blind, randomised, multicentre, multinational, phase 3a trial | None (treatment-naïve) | 388 | Semaglutide 0.5 mg OW s.c. Semaglutide 1.0 mg OW s.c. | Placebo | 30 weeks |
| **SUSTAIN 2** | NCT01930188 | Double-blind, randomised, multicentre, multinational, phase 3a trial | MET, TZD, or MET+TZD | 1231 | Semaglutide 0.5 mg OW s.c. + placebo Semaglutide 1.0 mg OW s.c. + placebo | Sitagliptin 100 mg p.o. OD + placebo | 56 weeks |
| **SUSTAIN 3** | NCT01885208 | Open-label, randomised, multicentre, multinational, phase 3a trial | One to two OADs of MET, TZD, or SU | 813 | Semaglutide 1.0 mg OW s.c. | Exenatide ER 2.0 mg OW s.c. | 56 weeks |
| **SUSTAIN 4** | NCT02128932 | Open-label, randomised, multicentre, multinational, phase 3a trial | MET or MET+SU | 1089 | Semaglutide 0.5 mg OW s.c. Semaglutide 1.0 mg OW s.c. | Insulin glargine starting from 10 IU OD | 30 weeks |
| **SUSTAIN 5** | NCT02305381 | Double-blind, randomised, multicentre, multinational, phase 3a trial | Basal insulin or basal  insulin+MET | 397 | Semaglutide 0.5 mg OW s.c. Semaglutide 1.0 mg OW s.c. | Placebo | 30 weeks |
| **SUSTAIN 7** | NCT02648204 | Open-label, randomised, multicentre, multinational, phase 3b trial | MET | 1201 | Semaglutide 0.5 mg OW s.c. Semaglutide 1.0 mg OW s.c. | Dulaglutide 0.75 mg OW s.c. Dulaglutide 1.5 mg OW s.c. | 40 weeks |
| **SUSTAIN 9** | NCT03086330 | Double-blind, randomised, multicentre, multinational, phase 3b trial | SGLT-2i, SGLT-2i+SU, or SGLT-2i+MET | 302 | Semaglutide 1.0 mg OW s.c. | Placebo | 30 weeks |
| **SUSTAIN 10** | NCT03191396 | Open-label, randomised, multicentre, multinational, phase 3b trial | One or more OADs of MET, SU, or SGLT-2i | 577 | Semaglutide 1.0 mg OW s.c. | Liraglutide 1.2 mg s.c. OD | 30 weeks |
| **SUSTAIN Japan OAD combination** | NCT02207374 | Open-label, randomised, multicentre, single-country, phase 3a trial | None or one OADs of α-GI, glinide, SU, or TZD | 601 | Semaglutide 0.5 mg OW s.c. Semaglutide 1.0 mg OW s.c. | Additional OAD (α-GI, MET, DPP-4 inhibitor, glinide, SU, or TZD) | 56 weeks |
| **SUSTAIN Japan Monotherapy** | NCT02254291 | Open-label, randomised, multicentre, single-country, phase 3a trial | None | 308 | Semaglutide 0.5 mg OW s.c. Semaglutide 1.0 mg OW s.c. | Sitagliptin 100 mg p.o. OD | 30 weeks |
| **SUSTAIN – China MRCT** | NCT03061214 | Double-blind, randomised, multicentre, multi-regional, phase 3a trial | MET | 868 | Semaglutide 0.5 mg OW s.c. + placebo Semaglutide 1.0 mg OW s.c. + placebo | Sitagliptin p.o. OD + placebo | 30 weeks |
| **PIONEER 1** | NCT02906930 | Double-blind, randomised, multicentre, multinational, phase 3a trial | None | 703 | Semaglutide 3 mg p.o. OD Semaglutide 7 mg p.o. OD Semaglutide 14 mg p.o. OD | Placebo | 26 weeks |
| **PIONEER 2** | NCT02863328 | Open-label, randomised, multicentre, multinational, phase 3a trial | MET | 822 | Semaglutide 14 mg p.o. OD | Empagliflozin 25 mg p.o. OD | 52 weeks |
| **PIONEER 4** | NCT02863419 | Double-blind, randomised, multicentre, multinational, phase 3a trial | MET or MET+SGLT-2i | 711 | Semaglutide 14 mg p.o. OD | Liraglutide 1.8 mg s.c. OD  Placebo | 52 weeks |
| **PIONEER 7** | NCT02849080 | Open-label, randomised, multicentre, multinational, phase 3a trial | One or two OAD of MET, SU, SGLT-2i, or TZD | 504 | Semaglutide flexible dose (3, 7, or 14 mg) p.o. OD | Sitagliptin 100 mg p.o. OD | 52 weeks |
| **PIONEER 8** | NCT03021187 | Double-blind, randomised, multicentre, multinational, phase 3a trial | Insulin (basal, basal-bolus, or premixed) or insulin (basal, basal-bolus, or premixed) + MET | 731 | Semaglutide 3 mg p.o. OD Semaglutide 7 mg p.o. OD Semaglutide 14 mg p.o. OD | Placebo | 52 weeks |

Abbreviations: α-GI, α-glucosidase inhibitor; DPP-4, dipeptidyl peptidase-4; eGFR, estimated glomerular filtration rate; exenatide ER, exenatide extended release; GLP-1RA, glucagon-like peptide-1 receptor agonist; HF, heart failure; MEN2, multiple endocrine neoplasia type 2; MET, metformin; MTC, medullary thyroid carcinoma; N, number of participants randomised; NYHA, New York Heart Association; OAD, oral antidiabetic drug; OD, once daily; OW, once weekly; p.o. per oral; s.c. subcutaneous; SU, sulphonylurea; T2D, type 2 diabetes; TZD, thiazolidinedione.

### Supplementary Table 3: Summary of Absolute Change in HbA_1c_ (%) by Trial

| Trial | Age group | Treatment | Estimated absolute change (%) (95% CI) |
| --- | --- | --- | --- |
| SUSTAIN 1 | ≤40 | Semaglutide 0.5 mg | -1.70 (-2.14 to -1.27) |
| SUSTAIN 1 | ≤40 | Semaglutide 1.0 mg | -1.86 (-2.18 to -1.55) |
| SUSTAIN 1 | ≤40 | Placebo | 0.68 (0.21 to 1.15) |
| SUSTAIN 1 | >40 and ≤50 | Semaglutide 0.5 mg | -1.74 (-2.00 to -1.49) |
| SUSTAIN 1 | >40 and ≤50 | Semaglutide 1.0 mg | -1.66 (-1.95 to -1.37) |
| SUSTAIN 1 | >40 and ≤50 | Placebo | -0.27 (-0.54 to 0.01) |
| SUSTAIN 1 | >50 | Semaglutide 0.5 mg | -1.38 (-1.55 to -1.20) |
| SUSTAIN 1 | >50 | Semaglutide 1.0 mg | -1.53 (-1.71 to -1.35) |
| SUSTAIN 1 | >50 | Placebo | -0.26 (-0.45 to -0.08) |
| SUSTAIN 2 | ≤40 | Semaglutide 0.5 mg | -1.26 (-1.49 to -1.02) |
| SUSTAIN 2 | ≤40 | Semaglutide 1.0 mg | -1.67 (-1.96 to -1.37) |
| SUSTAIN 2 | ≤40 | Sitagliptin 100 mg | -0.49 (-0.74 to -0.24) |
| SUSTAIN 2 | >40 and ≤50 | Semaglutide 0.5 mg | -1.49 (-1.63 to -1.35) |
| SUSTAIN 2 | >40 and ≤50 | Semaglutide 1.0 mg | -1.76 (-1.91 to -1.62) |
| SUSTAIN 2 | >40 and ≤50 | Sitagliptin 100 mg | -0.63 (-0.78 to -0.48) |
| SUSTAIN 2 | >50 | Semaglutide 0.5 mg | -1.41 (-1.50 to -1.33) |
| SUSTAIN 2 | >50 | Semaglutide 1.0 mg | -1.62 (-1.70 to -1.54) |
| SUSTAIN 2 | >50 | Sitagliptin 100 mg | -0.79 (-0.88 to -0.71) |
| SUSTAIN 3 | ≤40 | Semaglutide 1.0 mg | -1.43 (-1.70 to -1.15) |
| SUSTAIN 3 | ≤40 | Exenatide ER | -0.47 (-0.79 to -0.14) |
| SUSTAIN 3 | >40 and ≤50 | Semaglutide 1.0 mg | -1.54 (-1.73 to -1.35) |
| SUSTAIN 3 | >40 and ≤50 | Exenatide ER | -1.07 (-1.25 to -0.89) |
| SUSTAIN 3 | >50 | Semaglutide 1.0 mg | -1.72 (-1.81 to -1.62) |
| SUSTAIN 3 | >50 | Exenatide ER | -1.13 (-1.23 to -1.03) |
| SUSTAIN 4 | ≤40 | Semaglutide 0.5 mg | -0.97 (-1.25 to -0.68) |
| SUSTAIN 4 | ≤40 | Semaglutide 1.0 mg | -1.56 (-1.90 to -1.22) |
| SUSTAIN 4 | ≤40 | IGlar | -0.29 (-0.57 to -0.01) |
| SUSTAIN 4 | >40 and ≤50 | Semaglutide 0.5 mg | -1.24 (-1.41 to -1.07) |
| SUSTAIN 4 | >40 and ≤50 | Semaglutide 1.0 mg | -1.79 (-1.95 to -1.64) |
| SUSTAIN 4 | >40 and ≤50 | IGlar | -0.56 (-0.72 to -0.40) |
| SUSTAIN 4 | >50 | Semaglutide 0.5 mg | -1.27 (-1.36 to -1.18) |
| SUSTAIN 4 | >50 | Semaglutide 1.0 mg | -1.67 (-1.76 to -1.58) |
| SUSTAIN 4 | >50 | IGlar | -0.99 (-1.07 to -0.90) |
| SUSTAIN 5 | ≤40 | Semaglutide 0.5 mg | -1.22 (-1.81 to -0.62) |
| SUSTAIN 5 | ≤40 | Semaglutide 1.0 mg | -1.95 (-2.45 to -1.44) |
| SUSTAIN 5 | ≤40 | Placebo | 0.96 (0.41 to 1.51) |
| SUSTAIN 5 | >40 and ≤50 | Semaglutide 0.5 mg | -1.38 (-1.77 to -1.00) |
| SUSTAIN 5 | >40 and ≤50 | Semaglutide 1.0 mg | -2.03 (-2.40 to -1.65) |
| SUSTAIN 5 | >40 and ≤50 | Placebo | 0.08 (-0.24 to 0.40) |
| SUSTAIN 5 | >50 | Semaglutide 0.5 mg | -1.48 (-1.61 to -1.35) |
| SUSTAIN 5 | >50 | Semaglutide 1.0 mg | -1.86 (-1.99 to -1.72) |
| SUSTAIN 5 | >50 | Placebo | -0.27 (-0.42 to -0.13) |
| SUSTAIN 7 | ≤40 | Semaglutide 0.5 mg | -1.64 (-1.91 to -1.37) |
| SUSTAIN 7 | ≤40 | Semaglutide 1.0 mg | -1.77 (-2.02 to -1.51) |
| SUSTAIN 7 | ≤40 | Dulaglutide 0.75 mg | -1.07 (-1.33 to -0.81) |
| SUSTAIN 7 | ≤40 | Dulaglutide 1.5 mg | -1.22 (-1.47 to -0.97) |
| SUSTAIN 7 | >40 and ≤50 | Semaglutide 0.5 mg | -1.43 (-1.59 to -1.27) |
| SUSTAIN 7 | >40 and ≤50 | Semaglutide 1.0 mg | -1.92 (-2.09 to -1.74) |
| SUSTAIN 7 | >40 and ≤50 | Dulaglutide 0.75 mg | -1.02 (-1.18 to -0.86) |
| SUSTAIN 7 | >40 and ≤50 | Dulaglutide 1.5 mg | -1.21 (-1.38 to -1.05) |
| SUSTAIN 7 | >50 | Semaglutide 0.5 mg | -1.57 (-1.66 to -1.48) |
| SUSTAIN 7 | >50 | Semaglutide 1.0 mg | -1.81 (-1.90 to -1.71) |
| SUSTAIN 7 | >50 | Dulaglutide 0.75 mg | -1.21 (-1.30 to -1.12) |
| SUSTAIN 7 | >50 | Dulaglutide 1.5 mg | -1.49 (-1.58 to -1.40) |
| SUSTAIN 9 | ≤40 | Semaglutide 1.0 mg | -2.28 (-2.66 to -1.90) |
| SUSTAIN 9 | ≤40 | Placebo | 0.19 (-0.12 to 0.50) |
| SUSTAIN 9 | >40 and ≤50 | Semaglutide 1.0 mg | -1.58 (-1.83 to -1.34) |
| SUSTAIN 9 | >40 and ≤50 | Placebo | -0.04 (-0.25 to 0.18) |
| SUSTAIN 9 | >50 | Semaglutide 1.0 mg | -1.51 (-1.61 to -1.41) |
| SUSTAIN 9 | >50 | Placebo | -0.24 (-0.34 to -0.14) |
| SUSTAIN 10 | ≤40 | Semaglutide 1.0 mg | -1.69 (-1.99 to -1.40) |
| SUSTAIN 10 | ≤40 | Liraglutide 1.2 mg | -0.62 (-0.92 to -0.32) |
| SUSTAIN 10 | >40 and ≤50 | Semaglutide 1.0 mg | -2.04 (-2.21 to -1.88) |
| SUSTAIN 10 | >40 and ≤50 | Liraglutide 1.2 mg | -0.75 (-0.93 to -0.57) |
| SUSTAIN 10 | >50 | Semaglutide 1.0 mg | -1.66 (-1.73 to -1.58) |
| SUSTAIN 10 | >50 | Liraglutide 1.2 mg | -1.13 (-1.20 to -1.05) |
| SUSTAIN Japan OAD Combination | ≤40 | Semaglutide 0.5 mg | -1.34 (-1.64 to -1.03) |
| SUSTAIN Japan OAD Combination | ≤40 | Semaglutide 1.0 mg | -1.90 (-2.19 to -1.61) |
| SUSTAIN Japan OAD Combination | ≤40 | Additional OAD | 0.37 (-0.09 to 0.84) |
| SUSTAIN Japan OAD Combination | >40 and ≤50 | Semaglutide 0.5 mg | -1.56 (-1.71 to -1.42) |
| SUSTAIN Japan OAD Combination | >40 and ≤50 | Semaglutide 1.0 mg | -1.77 (-1.96 to -1.59) |
| SUSTAIN Japan OAD Combination | >40 and ≤50 | Additional OAD | -0.51 (-0.76 to -0.26) |
| SUSTAIN Japan OAD Combination | >50 | Semaglutide 0.5 mg | -1.82 (-1.90 to -1.74) |
| SUSTAIN Japan OAD Combination | >50 | Semaglutide 1.0 mg | -2.10 (-2.18 to -2.02) |
| SUSTAIN Japan OAD Combination | >50 | Additional OAD | -0.81 (-0.92 to -0.70) |
| SUSTAIN Japan Monotherapy | ≤40 | Semaglutide 0.5 mg | -1.67 (-2.13 to -1.21) |
| SUSTAIN Japan Monotherapy | ≤40 | Semaglutide 1.0 mg | -2.31 (-2.75 to -1.87) |
| SUSTAIN Japan Monotherapy | ≤40 | Sitagliptin 100 mg | -0.25 (-0.84 to 0.34) |
| SUSTAIN Japan Monotherapy | >40 and ≤50 | Semaglutide 0.5 mg | -1.94 (-2.21 to -1.68) |
| SUSTAIN Japan Monotherapy | >40 and ≤50 | Semaglutide 1.0 mg | -2.25 (-2.46 to -2.04) |
| SUSTAIN Japan Monotherapy | >40 and ≤50 | Sitagliptin 100 mg | -0.66 (-0.89 to -0.43) |
| SUSTAIN Japan Monotherapy | >50 | Semaglutide 0.5 mg | -1.88 (-2.00 to -1.77) |
| SUSTAIN Japan Monotherapy | >50 | Semaglutide 1.0 mg | -2.17 (-2.30 to -2.04) |
| SUSTAIN Japan Monotherapy | >50 | Sitagliptin 100 mg | -0.83 (-0.95 to -0.71) |
| SUSTAIN China MRCT | ≤40 | Semaglutide 0.5 mg | -1.42 (-1.62 to -1.22) |
| SUSTAIN China MRCT | ≤40 | Semaglutide 1.0 mg | -1.99 (-2.20 to -1.78) |
| SUSTAIN China MRCT | ≤40 | Sitagliptin 100 mg | -0.87 (-1.08 to -0.67) |
| SUSTAIN China MRCT | >40 and ≤50 | Semaglutide 0.5 mg | -1.55 (-1.73 to -1.38) |
| SUSTAIN China MRCT | >40 and ≤50 | Semaglutide 1.0 mg | -1.85 (-2.01 to -1.69) |
| SUSTAIN China MRCT | >40 and ≤50 | Sitagliptin 100 mg | -0.95 (-1.11 to -0.78) |
| SUSTAIN China MRCT | >50 | Semaglutide 0.5 mg | -1.42 (-1.51 to -1.32) |
| SUSTAIN China MRCT | >50 | Semaglutide 1.0 mg | -1.69 (-1.79 to -1.59) |
| SUSTAIN China MRCT | >50 | Sitagliptin 100 mg | -0.92 (-1.02 to -0.83) |
| PIONEER 1 | ≤40 | Oral semaglutide 3 mg | -0.98 (-1.35 to -0.61) |
| PIONEER 1 | ≤40 | Oral semaglutide 7 mg | -1.59 (-1.92 to -1.26) |
| PIONEER 1 | ≤40 | Oral semaglutide 14 mg | -1.81 (-2.20 to -1.42) |
| PIONEER 1 | ≤40 | Placebo | -0.65 (-1.09 to -0.21) |
| PIONEER 1 | >40 and ≤50 | Oral semaglutide 3 mg | -0.64 (-0.88 to -0.41) |
| PIONEER 1 | >40 and ≤50 | Oral semaglutide 7 mg | -1.31 (-1.60 to -1.02) |
| PIONEER 1 | >40 and ≤50 | Oral semaglutide 14 mg | -1.55 (-1.78 to -1.33) |
| PIONEER 1 | >40 and ≤50 | Placebo | -0.22 (-0.45 to 0.01) |
| PIONEER 1 | >50 | Oral semaglutide 3 mg | -0.96 (-1.11 to -0.81) |
| PIONEER 1 | >50 | Oral semaglutide 7 mg | -1.30 (-1.45 to -1.16) |
| PIONEER 1 | >50 | Oral semaglutide 14 mg | -1.50 (-1.65 to -1.34) |
| PIONEER 1 | >50 | Placebo | -0.24 (-0.39 to -0.08) |
| PIONEER 2 | ≤40 | Oral semaglutide 14 mg | -1.78 (-2.10 to -1.46) |
| PIONEER 2 | ≤40 | Empagliflozin 25 mg | -0.73 (-1.08 to -0.38) |
| PIONEER 2 | >40 and ≤50 | Oral semaglutide 14 mg | -1.36 (-1.53 to -1.18) |
| PIONEER 2 | >40 and ≤50 | Empagliflozin 25 mg | -0.97 (-1.14 to -0.80) |
| PIONEER 2 | >50 | Oral semaglutide 14 mg | -1.38 (-1.47 to -1.30) |
| PIONEER 2 | >50 | Empagliflozin 25 mg | -0.89 (-0.98 to -0.81) |
| PIONEER 4 | ≤40 | Oral semaglutide 14 mg | -1.46 (-1.84 to -1.08) |
| PIONEER 4 | ≤40 | Liraglutide 1.8 mg | -0.72 (-1.01 to -0.42) |
| PIONEER 4 | ≤40 | Placebo | 0.61 (-0.02 to 1.25) |
| PIONEER 4 | >40 and ≤50 | Oral semaglutide 14 mg | -1.09 (-1.26 to -0.92) |
| PIONEER 4 | >40 and ≤50 | Liraglutide 1.8 mg | -0.68 (-0.85 to -0.51) |
| PIONEER 4 | >40 and ≤50 | Placebo | -0.14 (-0.44 to 0.16) |
| PIONEER 4 | >50 | Oral semaglutide 14 mg | -1.31 (-1.41 to -1.22) |
| PIONEER 4 | >50 | Liraglutide 1.8 mg | -1.12 (-1.22 to -1.03) |
| PIONEER 4 | >50 | Placebo | -0.08 (-0.23 to 0.06) |
| PIONEER 7 | ≤40 | Oral semaglutide flex | -1.40 (-1.78 to -1.01) |
| PIONEER 7 | ≤40 | Sitagliptin 100 mg | -0.89 (-1.36 to -0.42) |
| PIONEER 7 | >40 and ≤50 | Oral semaglutide flex | -1.44 (-1.66 to -1.21) |
| PIONEER 7 | >40 and ≤50 | Sitagliptin 100 mg | -0.53 (-0.77 to -0.30) |
| PIONEER 7 | >50 | Oral semaglutide flex | -1.41 (-1.53 to -1.30) |
| PIONEER 7 | >50 | Sitagliptin 100 mg | -0.82 (-0.94 to -0.71) |
| PIONEER 8 | ≤40 | Oral semaglutide 3 mg | 0.11 (-0.54 to 0.76) |
| PIONEER 8 | ≤40 | Oral semaglutide 7 mg | -0.48 (-1.39 to 0.43) |
| PIONEER 8 | ≤40 | Oral semaglutide 14 mg | -0.37 (-1.33 to 0.59) |
| PIONEER 8 | ≤40 | Placebo | 0.20 (-0.63 to 1.03) |
| PIONEER 8 | >40 and ≤50 | Oral semaglutide 3 mg | -0.53 (-0.96 to -0.10) |
| PIONEER 8 | >40 and ≤50 | Oral semaglutide 7 mg | -0.59 (-0.97 to -0.22) |
| PIONEER 8 | >40 and ≤50 | Oral semaglutide 14 mg | -1.18 (-1.51 to -0.84) |
| PIONEER 8 | >40 and ≤50 | Placebo | 0.04 (-0.35 to 0.44) |
| PIONEER 8 | >50 | Oral semaglutide 3 mg | -0.61 (-0.75 to -0.47) |
| PIONEER 8 | >50 | Oral semaglutide 7 mg | -0.96 (-1.09 to -0.82) |
| PIONEER 8 | >50 | Oral semaglutide 14 mg | -1.35 (-1.49 to -1.21) |
| PIONEER 8 | >50 | Placebo | -0.12 (-0.26 to 0.02) |

Abbreviations: CI, confidence interval; IGlar, insulin glargine; MRCT, multi-regional clinical trial; OAD, oral anti-diabetic

### Supplementary Table 4: Summary of HbA_1c_ Endpoints

| Trial Name | Comparison | Age Group | ETD (95% CI) | P interaction value |
| --- | --- | --- | --- | --- |
| SUSTAIN 1 | Semaglutide 1.0 mg vs Placebo | ≤40 | -2.5 (-3.1 to -2.0) | 0.001 |
|  |  | >40 and ≤50 | -1.4 (-1.8 to -1.0) |  |
|  |  | >50 | -1.3 (-1.5 to -1.0) |  |
| SUSTAIN 9 | Semaglutide 1.0 mg vs Placebo | ≤40 | -2.5 (-3.0 to -2.0) | 0.002 |
|  |  | >40 and ≤50 | -1.5 (-1.9 to -1.2) |  |
|  |  | >50 | -1.3 (-1.4 to -1.1) |  |
| SUSTAIN 5 | Semaglutide 1.0 mg vs Placebo | ≤40 | -2.9 (-3.7 to -2.2) | 0.08 |
|  |  | >40 and ≤50 | -2.1 (-2.6 to -1.6) |  |
|  |  | >50 | -1.6 (-1.8 to -1.4) |  |
| SUSTAIN 2 | Semaglutide 1.0 mg vs Sitagliptin 100 mg | ≤40 | -1.2 (-1.6 to -0.8) | 0.83 |
|  |  | >40 and ≤50 | -1.1 (-1.3 to -0.9) |  |
|  |  | >50 | -0.8 (-0.9 to -0.7) |  |
| SUSTAIN Japan Monotherapy | Semaglutide 1.0 mg vs Sitagliptin 100 mg | ≤40 | -2.1 (-2.8 to -1.3) | 0.25 |
|  |  | >40 and ≤50 | -1.6 (-1.9 to -1.3) |  |
|  |  | >50 | -1.3 (-1.5 to -1.2) |  |
| SUSTAIN China MRCT | Semaglutide 1.0 mg vs Sitagliptin 100 mg | ≤40 | -1.1 (-1.4 to -0.8) | 0.25 |
|  |  | >40 and ≤50 | -0.9 (-1.1 to -0.7) |  |
|  |  | >50 | -0.8 (-0.9 to -0.6) |  |
| SUSTAIN 10 | Semaglutide 1.0 mg vs Liraglutide 1.2 mg | ≤40 | -1.1 (-1.5 to -0.7) | 0.38 |
|  |  | >40 and ≤50 | -1.3 (-1.5 to -1.1) |  |
|  |  | >50 | -0.5 (-0.6 to -0.4) |  |
| SUSTAIN 4 | Semaglutide 1.0 mg vs Insulin Glargine | ≤40 | -1.3 (-1.7 to -0.8) | 0.88 |
|  |  | >40 and ≤50 | -1.2 (-1.5 to -1.0) |  |
|  |  | >50 | -0.7 (-0.8 to -0.6) |  |
| SUSTAIN 3 | Semaglutide 1.0 mg vs Exenatide ER | ≤40 | -1.0 (-1.4 to -0.5) | 0.06 |
|  |  | >40 and ≤50 | -0.5 (-0.7 to -0.2) |  |
|  |  | >50 | -0.6 (-0.7 to -0.5) |  |
| SUSTAIN 7 | Semaglutide 1.0 mg vs Dulaglutide 1.5 mg | ≤40 | -0.5 (-0.9 to -0.2) | 0.47 |
|  |  | >40 and ≤50 | -0.7 (-0.9 to -0.5) |  |
|  |  | >50 | -0.3 (-0.4 to -0.2) |  |
| SUSTAIN Japan OAD Combination | Semaglutide 1.0 mg vs OAD monotherapy + diet/exercise | ≤40 | -2.3 (-2.8 to -1.7) | 0.002 |
|  |  | >40 and ≤50 | -1.3 (-1.6 to -1.0) |  |
|  |  | >50 | -1.3 (-1.4 to -1.2) |  |
| SUSTAIN 1 | Semaglutide 0.5 mg vs Placebo | ≤40 | -2.4 (-3.0 to -1.7) | 0.02 |
|  |  | >40 and ≤50 | -1.5 (-1.9 to -1.1) |  |
|  |  | >50 | -1.1 (-1.4 to -0.9) |  |
| SUSTAIN 5 | Semaglutide 0.5 mg vs Placebo | ≤40 | -2.2 (-3.0 to -1.4) | 0.14 |
|  |  | >40 and ≤50 | -1.5 (-2.0 to -1.0) |  |
|  |  | >50 | -1.2 (-1.4 to -1.0) |  |
| SUSTAIN 2 | Semaglutide 0.5 mg vs Sitagliptin 100 mg | ≤40 | -0.8 (-1.1 to -0.4) | 0.66 |
|  |  | >40 and ≤50 | -0.9 (-1.1 to -0.7) |  |
|  |  | >50 | -0.6 (-0.7 to -0.5) |  |
| SUSTAIN Japan Monotherapy | Semaglutide 0.5 mg vs Sitagliptin 100 mg | ≤40 | -1.4 (-2.2 to -0.7) | 0.74 |
|  |  | >40 and ≤50 | -1.3 (-1.6 to -0.9) |  |
|  |  | >50 | -1.1 (-1.2 to -0.9) |  |
| SUSTAIN China MRCT | Semaglutide 0.5 mg vs Sitagliptin 100 mg | ≤40 | -0.5 (-0.8 to -0.3) | 0.77 |
|  |  | >40 and ≤50 | -0.6 (-0.8 to -0.4) |  |
|  |  | >50 | -0.5 (-0.6 to -0.4) |  |
| SUSTAIN 4 | Semaglutide 0.5 mg vs Insulin Glargine | ≤40 | -0.7 (-1.1 to -0.3) | 0.99 |
|  |  | >40 and ≤50 | -0.7 (-0.9 to -0.4) |  |
|  |  | >50 | -0.3 (-0.4 to -0.2) |  |
| SUSTAIN 7 | Semaglutide 0.5 mg vs Dulaglutide 0.75 mg | ≤40 | -0.6 (-0.9 to -0.2) | 0.48 |
|  |  | >40 and ≤50 | -0.4 (-0.6 to -0.2) |  |
|  |  | >50 | -0.4 (-0.5 to -0.2) |  |
| SUSTAIN Japan OAD Combination | Semaglutide 0.5 mg vs OAD monotherapy + diet/exercise | ≤40 | -1.7 (-2.3 to -1.1) | 0.04 |
|  |  | >40 and ≤50 | -1.1 (-1.3 to -0.8) |  |
|  |  | >50 | -1.0 (-1.1 to -0.9) |  |
| PIONEER 2 | Semaglutide 3 mg vs Placebo | ≤40 | -0.3 (-0.9 to 0.2) | 0.78 |
|  |  | >40 and ≤50 | -0.4 (-0.8 to -0.1) |  |
|  |  | >50 | -0.7 (-0.9 to -0.5) |  |
| PIONEER 8 | Semaglutide 3 mg vs Placebo | ≤40 | -0.1 (-1.1 to 1.0) | 0.43 |
|  |  | >40 and ≤50 | -0.6 (-1.2 to 0.0) |  |
|  |  | >50 | -0.5 (-0.7 to -0.3) |  |
| PIONEER 1 | Semaglutide 7 mg vs Placebo | ≤40 | -0.9 (-1.5 to -0.4) | 0.68 |
|  |  | >40 and ≤50 | -1.1 (-1.5 to -0.7) |  |
|  |  | >50 | -1.1 (-1.3 to -0.9) |  |
| PIONEER 8 | Semaglutide 7 mg vs Placebo | ≤40 | -0.7 (-1.9 to 0.6) | 0.96 |
|  |  | >40 and ≤50 | -0.6 (-1.2 to -0.1) |  |
|  |  | >50 | -0.8 (-1.0 to -0.6) |  |
| PIONEER 4 | Semaglutide 14 mg vs Placebo | ≤40 | -2.1 (-2.8 to -1.3) | 0.007 |
|  |  | >40 and ≤50 | -1.0 (-1.3 to -0.6) |  |
|  |  | >50 | -1.2 (-1.4 to -1.1) |  |
| PIONEER 1 | Semaglutide 14 mg vs Placebo | ≤40 | -1.2 (-1.8 to -0.6) | 0.62 |
|  |  | >40 and ≤50 | -1.3 (-1.6 to -1.0) |  |
|  |  | >50 | -1.3 (-1.5 to -1.0) |  |
| PIONEER 8 | Semaglutide 14 mg vs Placebo | ≤40 | -0.6 (-1.8 to 0.7) | 0.35 |
|  |  | >40 and ≤50 | -1.2 (-1.7 to -0.7) |  |
|  |  | >50 | -1.2 (-1.4 to -1.0) |  |
| PIONEER 2 | Semaglutide 14 mg vs Empagliflozin 25 mg | ≤40 | -1.1 (-1.5 to -0.6) | 0.01 |
|  |  | >40 and ≤50 | -0.4 (-0.6 to -0.1) |  |
|  |  | >50 | -0.5 (-0.6 to -0.4) |  |
| PIONEER 4 | Semaglutide 14 mg vs Liraglutide 1.8 mg | ≤40 | -0.7 (-1.2 to -0.3) | 0.23 |
|  |  | >40 and ≤50 | -0.4 (-0.7 to -0.2) |  |
|  |  | >50 | -0.2 (-0.3 to -0.1) |  |
| PIONEER 7 | Semaglutide Flex vs Sitagliptin 100 mg | ≤40 | -0.5 (-1.1 to 0.1) | 0.27 |
|  |  | >40 and ≤50 | -0.9 (-1.2 to -0.6) |  |
|  |  | >50 | -0.6 (-0.7 to -0.4) |  |

Abbreviations: CI, confidence interval; MRCT, multi-regional clinical trial; OAD, oral anti-diabetic

### Supplementary Table 5: Summary of Absolute Change in Body Weight (kg) by Trial

| Trial | Age group | Treatment | Estimated absolute change (kg) (95% CI) |
| --- | --- | --- | --- |
| SUSTAIN 1 | ≤40 | Semaglutide 0.5 mg | -2.96 (-4.58 to -1.35) |
| SUSTAIN 1 | ≤40 | Semaglutide 1.0 mg | -3.72 (-4.96 to -2.48) |
| SUSTAIN 1 | ≤40 | Placebo | 0.41(-1.36 to 2.18) |
| SUSTAIN 1 | >40 and ≤50 | Semaglutide 0.5 mg | -3.76 (-4.76 to -2.76) |
| SUSTAIN 1 | >40 and ≤50 | Semaglutide 1.0 mg | -3.11 (-4.23 to -1.99) |
| SUSTAIN 1 | >40 and ≤50 | Placebo | -1.51 (-2.56 to -0.46) |
| SUSTAIN 1 | >50 | Semaglutide 0.5 mg | -4.02 (-4.72 to -3.33) |
| SUSTAIN 1 | >50 | Semaglutide 1.0 mg | -5.24 (-5.94 to -4.54) |
| SUSTAIN 1 | >50 | Placebo | -1.87 (-2.61 to -1.14) |
| SUSTAIN 2 | ≤40 | Semaglutide 0.5 mg | -3.17 (-4.32 to -2.02) |
| SUSTAIN 2 | ≤40 | Semaglutide 1.0 mg | -5.48 (-6.94 to -4.03) |
| SUSTAIN 2 | ≤40 | Sitagliptin 100 mg | -4.10 (-5.34 to -2.85) |
| SUSTAIN 2 | >40 and ≤50 | Semaglutide 0.5 mg | -3.93 (-4.62 to -3.23) |
| SUSTAIN 2 | >40 and ≤50 | Semaglutide 1.0 mg | -6.03 (-6.74 to -5.31) |
| SUSTAIN 2 | >40 and ≤50 | Sitagliptin 100 mg | -1.77 (-2.49 to -1.05) |
| SUSTAIN 2 | >50 | Semaglutide 0.5 mg | -4.72 (-5.14 to -4.30) |
| SUSTAIN 2 | >50 | Semaglutide 1.0 mg | -6.27 (-6.68 to -5.86) |
| SUSTAIN 2 | >50 | Sitagliptin 100 mg | -1.85 (-2.28 to -1.41) |
| SUSTAIN 3 | ≤40 | Semaglutide 1.0 mg | -4.73 (-6.06 to -3.39) |
| SUSTAIN 3 | ≤40 | Exenatide ER | -1.24 (-2.82 to 0.34) |
| SUSTAIN 3 | >40 and ≤50 | Semaglutide 1.0 mg | -4.51 (-5.40 to -3.62) |
| SUSTAIN 3 | >40 and ≤50 | Exenatide ER | -1.82 (-2.68 to -0.97) |
| SUSTAIN 3 | >50 | Semaglutide 1.0 mg | -6.22 (-6.66 to -5.78) |
| SUSTAIN 3 | >50 | Exenatide ER | -2.03 (-2.48 to -1.58) |
| SUSTAIN 4 | ≤40 | Semaglutide 0.5 mg | -2.09 (-3.24 to -0.94) |
| SUSTAIN 4 | ≤40 | Semaglutide 1.0 mg | -3.81 (-5.2 to -2.42) |
| SUSTAIN 4 | ≤40 | IGlar | 1.67 (0.53 to 2.80) |
| SUSTAIN 4 | >40 and ≤50 | Semaglutide 0.5 mg | -3.91 (-4.59 to -3.22) |
| SUSTAIN 4 | >40 and ≤50 | Semaglutide 1.0 mg | -5.75 (-6.39 to -5.10) |
| SUSTAIN 4 | >40 and ≤50 | IGlar | 1.27 (0.61 to 1.92) |
| SUSTAIN 4 | >50 | Semaglutide 0.5 mg | -3.51 (-3.87 to -3.15) |
| SUSTAIN 4 | >50 | Semaglutide 1.0 mg | -5.12 (-5.49 to -4.75) |
| SUSTAIN 4 | >50 | IGlar | 1.03 (0.68 to 1.38) |
| SUSTAIN 5 | ≤40 | Semaglutide 0.5 mg | -2.10 (-4.47 to 0.26) |
| SUSTAIN 5 | ≤40 | Semaglutide 1.0 mg | -5.56 (-7.60 to -3.52) |
| SUSTAIN 5 | ≤40 | Placebo | -0.17 (-2.38 to 2.04) |
| SUSTAIN 5 | >40 and ≤50 | Semaglutide 0.5 mg | -2.56 (-4.10 to -1.01) |
| SUSTAIN 5 | >40 and ≤50 | Semaglutide 1.0 mg | -6.94 (-8.45 to -5.42) |
| SUSTAIN 5 | >40 and ≤50 | Placebo | 0.11 (-1.17 to 1.39) |
| SUSTAIN 5 | >50 | Semaglutide 0.5 mg | -3.86 (-4.39 to -3.34) |
| SUSTAIN 5 | >50 | Semaglutide 1.0 mg | -6.44 (-6.97 to -5.9) |
| SUSTAIN 5 | >50 | Placebo | -1.60 (-2.17 to -1.02) |
| SUSTAIN 7 | ≤40 | Semaglutide 0.5 mg | -3.76 (-5.00 to -2.52) |
| SUSTAIN 7 | ≤40 | Semaglutide 1.0 mg | -6.80 (-7.96 to -5.65) |
| SUSTAIN 7 | ≤40 | Dulaglutide 0.75 mg | -2.31 (-3.49 to -1.13) |
| SUSTAIN 7 | ≤40 | Dulaglutide 1.5 mg | -2.97 (-4.10 to -1.84) |
| SUSTAIN 7 | >40 and ≤50 | Semaglutide 0.5 mg | -4.37 (-5.09 to -3.64) |
| SUSTAIN 7 | >40 and ≤50 | Semaglutide 1.0 mg | -6.76 (-7.55 to -5.98) |
| SUSTAIN 7 | >40 and ≤50 | Dulaglutide 0.75 mg | -2.06 (-2.78 to -1.35) |
| SUSTAIN 7 | >40 and ≤50 | Dulaglutide 1.5 mg | -2.92 (-3.66 to -2.17) |
| SUSTAIN 7 | >50 | Semaglutide 0.5 mg | -4.69 (-5.11 to -4.28) |
| SUSTAIN 7 | >50 | Semaglutide 1.0 mg | -6.65 (-7.06 to -6.24) |
| SUSTAIN 7 | >50 | Dulaglutide 0.75 mg | -2.26 (-2.67 to -1.85) |
| SUSTAIN 7 | >50 | Dulaglutide 1.5 mg | -3.20 (-3.61 to -2.78) |
| SUSTAIN 9 | ≤40 | Semaglutide 1.0 mg | -7.90 (-9.46 to -6.35) |
| SUSTAIN 9 | ≤40 | Placebo | -0.84 (-2.17 to 0.50) |
| SUSTAIN 9 | >40 and ≤50 | Semaglutide 1.0 mg | -4.38 (-5.44 to -3.31) |
| SUSTAIN 9 | >40 and ≤50 | Placebo | 0.35 (-0.59 to 1.28) |
| SUSTAIN 9 | >50 | Semaglutide 1.0 mg | -4.57 (-5.00 to -4.14) |
| SUSTAIN 9 | >50 | Placebo | -1.24 (-1.68 to -0.81) |
| SUSTAIN 10 | ≤40 | Semaglutide 1.0 mg | -5.78 (-7.21 to -4.36) |
| SUSTAIN 10 | ≤40 | Liraglutide 1.2 mg | -2.85 (-4.33 to -1.37) |
| SUSTAIN 10 | >40 and ≤50 | Semaglutide 1.0 mg | -7.26 (-8.08 to -6.44) |
| SUSTAIN 10 | >40 and ≤50 | Liraglutide 1.2 mg | -0.71 (-1.56 to 0.15) |
| SUSTAIN 10 | >50 | Semaglutide 1.0 mg | -5.43 (-5.80 to -5.06) |
| SUSTAIN 10 | >50 | Liraglutide 1.2 mg | -2.11 (-2.47 to -1.74) |
| SUSTAIN Japan OAD Combination | ≤40 | Semaglutide 0.5 mg | -0.79 (-2.36 to 0.79) |
| SUSTAIN Japan OAD Combination | ≤40 | Semaglutide 1.0 mg | -4.66 (-6.15 to -3.17) |
| SUSTAIN Japan OAD Combination | ≤40 | Additional OAD | 0.67 (-1.66 to 3.00) |
| SUSTAIN Japan OAD Combination | >40 and ≤50 | Semaglutide 0.5 mg | -0.83 (-1.59 to -0.07) |
| SUSTAIN Japan OAD Combination | >40 and ≤50 | Semaglutide 1.0 mg | -1.36 (-2.29 to -0.43) |
| SUSTAIN Japan OAD Combination | >40 and ≤50 | Additional OAD | -0.21 (-1.45 to 1.04) |
| SUSTAIN Japan OAD Combination | >50 | Semaglutide 0.5 mg | -1.58 (-1.97 to -1.18) |
| SUSTAIN Japan OAD Combination | >50 | Semaglutide 1.0 mg | -3.23 (-3.62 to -2.85) |
| SUSTAIN Japan OAD Combination | >50 | Additional OAD | 0.43 (-0.11 to 0.96) |
| SUSTAIN Japan Monotherapy | ≤40 | Semaglutide 0.5 mg | -2.93 (-4.60 to -1.27) |
| SUSTAIN Japan Monotherapy | ≤40 | Semaglutide 1.0 mg | -6.61 (-8.29 to -4.94) |
| SUSTAIN Japan Monotherapy | ≤40 | Sitagliptin 100 mg | -1.58 (-3.76 to 0.60) |
| SUSTAIN Japan Monotherapy | >40 and ≤50 | Semaglutide 0.5 mg | -1.13 (-2.10 to -0.17) |
| SUSTAIN Japan Monotherapy | >40 and ≤50 | Semaglutide 1.0 mg | -2.51 (-3.32 to -1.71) |
| SUSTAIN Japan Monotherapy | >40 and ≤50 | Sitagliptin 100 mg | 0.90 (0.07 to 1.72) |
| SUSTAIN Japan Monotherapy | >50 | Semaglutide 0.5 mg | -2.31 (-2.73 to -1.89) |
| SUSTAIN Japan Monotherapy | >50 | Semaglutide 1.0 mg | -4.07 (-4.53 to -3.61) |
| SUSTAIN Japan Monotherapy | >50 | Sitagliptin 100 mg | -0.07 (-0.50 to 0.36) |
| SUSTAIN China MRCT | ≤40 | Semaglutide 0.5 mg | -3.08 (-3.80 to -2.35) |
| SUSTAIN China MRCT | ≤40 | Semaglutide 1.0 mg | -5.04 (-5.79 to -4.28) |
| SUSTAIN China MRCT | ≤40 | Sitagliptin 100 mg | 0.23 (-0.48 to 0.95) |
| SUSTAIN China MRCT | >40 and ≤50 | Semaglutide 0.5 mg | -2.30 (-2.92 to -1.69) |
| SUSTAIN China MRCT | >40 and ≤50 | Semaglutide 1.0 mg | -3.49 (-4.04 to -2.94) |
| SUSTAIN China MRCT | >40 and ≤50 | Sitagliptin 100 mg | -0.87 (-1.45 to -0.29) |
| SUSTAIN China MRCT | >50 | Semaglutide 0.5 mg | -3.12 (-3.47 to -2.77) |
| SUSTAIN China MRCT | >50 | Semaglutide 1.0 mg | -4.25 (-4.61 to -3.89) |
| SUSTAIN China MRCT | >50 | Sitagliptin 100 mg | -0.57 (-0.91 to -0.22) |
| PIONEER 1 | ≤40 | Oral semaglutide 3 mg | -2.08 (-3.27 to -0.90) |
| PIONEER 1 | ≤40 | Oral semaglutide 7 mg | -3.19 (-4.27 to -2.10) |
| PIONEER 1 | ≤40 | Oral semaglutide 14 mg | -5.92 (-7.20 to -4.65) |
| PIONEER 1 | ≤40 | Placebo | -0.43 (-1.88 to 1.03) |
| PIONEER 1 | >40 and ≤50 | Oral semaglutide 3 mg | -2.13 (-2.90 to -1.36) |
| PIONEER 1 | >40 and ≤50 | Oral semaglutide 7 mg | -2.82 (-3.75 to -1.88) |
| PIONEER 1 | >40 and ≤50 | Oral semaglutide 14 mg | -3.18 (-3.90 to -2.45) |
| PIONEER 1 | >40 and ≤50 | Placebo | -1.46 (-2.20 to -0.72) |
| PIONEER 1 | >50 | Oral semaglutide 3 mg | -1.62 (-2.11 to -1.14) |
| PIONEER 1 | >50 | Oral semaglutide 7 mg | -2.62 (-3.09 to -2.16) |
| PIONEER 1 | >50 | Oral semaglutide 14 mg | -4.46 (-4.95 to -3.96) |
| PIONEER 1 | >50 | Placebo | -1.64 (-2.14 to -1.13) |
| PIONEER 2 | ≤40 | Oral semaglutide 14 mg | -6.23 (-7.80 to -4.66) |
| PIONEER 2 | ≤40 | Empagliflozin 25 mg | -3.10 (-4.78 to -1.42) |
| PIONEER 2 | >40 and ≤50 | Oral semaglutide 14 mg | -3.89 (-4.73 to -3.04) |
| PIONEER 2 | >40 and ≤50 | Empagliflozin 25 mg | -3.65 (-4.49 to -2.80) |
| PIONEER 2 | >50 | Oral semaglutide 14 mg | -4.84 (-5.25 to -4.43) |
| PIONEER 2 | >50 | Empagliflozin 25 mg | -4.06 (-4.46 to -3.66) |
| PIONEER 4 | ≤40 | Oral semaglutide 14 mg | -3.81 (-5.68 to -1.94) |
| PIONEER 4 | ≤40 | Liraglutide 1.8 mg | -1.81 (-3.27 to -0.35) |
| PIONEER 4 | ≤40 | Placebo | -2.25 (-5.37 to 0.87) |
| PIONEER 4 | >40 and ≤50 | Oral semaglutide 14 mg | -4.02 (-4.85 to -3.19) |
| PIONEER 4 | >40 and ≤50 | Liraglutide 1.8 mg | -2.47 (-3.34 to -1.61) |
| PIONEER 4 | >40 and ≤50 | Placebo | -1.18 (-2.60 to 0.25) |
| PIONEER 4 | >50 | Oral semaglutide 14 mg | -5.41 (-5.88 to -4.93) |
| PIONEER 4 | >50 | Liraglutide 1.8 mg | -3.62 (-4.09 to -3.15) |
| PIONEER 4 | >50 | Placebo | -1.44 (-2.14 to -0.74) |
| PIONEER 7 | Age <= 40 | Oral semaglutide flex | -2.07 (-3.52 to -0.61) |
| PIONEER 7 | Age <= 40 | Sitagliptin 100 mg | -0.97 (-2.76 to 0.81) |
| PIONEER 7 | >40 and ≤50 | Oral semaglutide flex | -2.32 (-3.17 to -1.48) |
| PIONEER 7 | >40 and ≤50 | Sitagliptin 100 mg | -0.73 (-1.62 to 0.16) |
| PIONEER 7 | >50 | Oral semaglutide flex | -3.13 (-3.55 to -2.70) |
| PIONEER 7 | >50 | Sitagliptin 100 mg | -0.90 (-1.33 to -0.48) |
| PIONEER 8 | ≤40 | Oral semaglutide 3 mg | 0.73 (-1.62 to 3.08) |
| PIONEER 8 | ≤40 | Oral semaglutide 7 mg | 1.57 (-1.74 to 4.88) |
| PIONEER 8 | ≤40 | Oral semaglutide 14 mg | -2.20 (-5.69 to 1.29) |
| PIONEER 8 | ≤40 | Placebo | 2.72 (-0.31 to 5.76) |
| PIONEER 8 | >40 and ≤50 | Oral semaglutide 3 mg | -0.39 (-1.91 to 1.14) |
| PIONEER 8 | >40 and ≤50 | Oral semaglutide 7 mg | -2.46 (-3.81 to -1.10) |
| PIONEER 8 | >40 and ≤50 | Oral semaglutide 14 mg | -4.69 (-5.90 to -3.48) |
| PIONEER 8 | >40 and ≤50 | Placebo | 0.67 (-0.75 to 2.10) |
| PIONEER 8 | >50 | Oral semaglutide 3 mg | -1.49 (-1.98 to -0.99) |
| PIONEER 8 | >50 | Oral semaglutide 7 mg | -3.27 (-3.75 to -2.78) |
| PIONEER 8 | >50 | Oral semaglutide 14 mg | -4.38 (-4.88 to -3.88) |
| PIONEER 8 | >50 | Placebo | 0.38 (-0.13 to 0.89) |

Abbreviations: CI, confidence interval; IGlar, insulin glargine; MRCT, multi-regional clinical trial; OAD, oral anti-diabetic

### Supplementary Table 6: Summary of Body Weight Endpoints

| Trial Name | Comparison | Age Group | ETD (95% CI) | P interaction value |
| --- | --- | --- | --- | --- |
| SUSTAIN 1 | Semaglutide 1.0 mg vs Placebo | ≤40 | -4.1 (-6.3 to -2.0) | 0.06 |
|  |  | >40 and ≤50 | -1.6 (-3.1 to -0.1) |  |
|  |  | >50 | -3.4 (-4.4 to -2.4) |  |
| SUSTAIN 9 | Semaglutide 1.0 mg vs Placebo | ≤40 | -7.1 (-9.1 to -5.0) | 0.07 |
|  |  | >40 and ≤50 | -4.7 (-6.1 to -3.3) |  |
|  |  | >50 | -3.3 (-3.9 to -2.7) |  |
| SUSTAIN 5 | Semaglutide 1.0 mg vs Placebo | ≤40 | -5.4 (-8.4 to -2.4) | 0.37 |
|  |  | >40 and ≤50 | -7.0 (-9.0 to -5.1) |  |
|  |  | >50 | -4.8 (-5.6 to -4.1) |  |
| SUSTAIN 1 | Semaglutide 0.5 mg vs Placebo | ≤40 | -3.4 (-5.8 to -1.0) | 0.43 |
|  |  | >40 and ≤50 | -2.2 (-3.7 to -0.8) |  |
|  |  | >50 | -2.2 (-3.2 to -1.1) |  |
| SUSTAIN 5 | Semaglutide 0.5 mg vs Placebo | ≤40 | -1.9 (-5.2 to 1.3) | 0.71 |
|  |  | >40 and ≤50 | -2.7 (-4.7 to -0.7) |  |
|  |  | >50 | -2.3 (-3.0 to -1.5) |  |
| SUSTAIN 2 | Semaglutide 1.0 mg vs Sitagliptin 100 mg | ≤40 | -1.4 (-3.3 to 0.5) | 0.01 |
|  |  | >40 and ≤50 | -4.3 (-5.3 to -3.2) |  |
|  |  | >50 | -4.4 (-5 to -3.8) |  |
| SUSTAIN Japan Monotherapy | Semaglutide 1.0 mg vs Sitagliptin 100 mg | ≤40 | -5.0 (-7.7 to -2.4) | 0.27 |
|  |  | >40 and ≤50 | -3.4 (-4.5 to -2.3) |  |
|  |  | >50 | -4.0 (-4.6 to -3.4) |  |
| SUSTAIN China MRCT | Semaglutide 1.0 mg vs Sitagliptin 100 mg | ≤40 | -5.3 (-6.3 to -4.2) | <0.0001 |
|  |  | >40 and ≤50 | -2.6 (-3.4 to -1.8) |  |
|  |  | >50 | -3.7 (-4.2 to -3.2) |  |
| SUSTAIN 2 | Semaglutide 0.5 mg vs Sitagliptin 100 mg | ≤40 | 0.9 (-0.8 to 2.6) | 0.002 |
|  |  | >40 and ≤50 | -2.2 (-3.2 to -1.2) |  |
|  |  | >50 | -2.9 (-3.5 to -2.3) |  |
| SUSTAIN Japan Monotherapy | Semaglutide 0.5 mg vs Sitagliptin 100 mg | ≤40 | -1.4 (-4.1to 1.4) | 0.66 |
|  |  | >40 and ≤50 | -2.0 (-3.3 to -0.8) |  |
|  |  | >50 | -2.2 (-2.8 to -1.6) |  |
| SUSTAIN China MRCT | Semaglutide 0.5 mg vs Sitagliptin 100 mg | ≤40 | -3.3 (-4.3 to -2.3) | 0.005 |
|  |  | >40 and ≤50 | -1.4 (-2.3 to -0.6) |  |
|  |  | >50 | -2.6 (-3.0 to -2.1) |  |
| SUSTAIN 10 | Semaglutide 1.0 mg vs Liraglutide 1.2 mg | ≤40 | -2.9 (-5.0 to -0.9) | 0.03 |
|  |  | >40 and ≤50 | -6.6 (-7.7 to -5.4) |  |
|  |  | >50 | -3.3 (-3.8 to -2.8) |  |
| SUSTAIN 4 | Semaglutide 1.0 mg vs Insulin Glargine | ≤40 | -5.5 (-7.3 to -3.7) | 0.14 |
|  |  | >40 and ≤50 | -7.0 (-7.9 to -6.1) |  |
|  |  | >50 | -6.1 (-6.7 to -5.6) |  |
| SUSTAIN 4 | Semaglutide 0.5 mg vs Insulin Glargine | ≤40 | -3.8 (-5.4 to -2.1) | 0.14 |
|  |  | >40 and ≤50 | -5.2 (-6.1 to -4.2) |  |
|  |  | >50 | -4.5 (-5.0 to -4.0) |  |
| SUSTAIN 3 | Semaglutide 1.0 mg vs Exenatide ER | ≤40 | -3.5 (-5.5 to -1.4) | 0.52 |
|  |  | >40 and ≤50 | -2.7 (-3.9 to -1.5) |  |
|  |  | >50 | -4.2 (-4.8 to -3.6) |  |
| SUSTAIN 7 | Semaglutide 1.0 mg vs Dulaglutide 1.5 mg | ≤40 | -3.8 (-5.4 to -2.2) | 0.99 |
|  |  | >40 and ≤50 | -3.8 (-4.9 to -2.8) |  |
|  |  | >50 | -3.5 (-4.0 to -2.9) |  |
| SUSTAIN 7 | Semaglutide 0.5 mg vs Dulaglutide 0.75 mg | ≤40 | -1.4 (-3.2 to 0.3) | 0.40 |
|  |  | >40 and ≤50 | -2.3 (-3.3 to -1.3) |  |
|  |  | >50 | -2.4 (-3.0 to -1.8) |  |
| SUSTAIN Japan OAD combination | Semaglutide 1.0 mg vs OAD monotherapy + diet/exercise | ≤40 | -5.3 (-8.1 to -2.6) | 0.009 |
|  |  | >40 and ≤50 | -1.2 (-2.7 to 0.4) |  |
|  |  | >50 | -3.7 (-4.3 to -3.0) |  |
| SUSTAIN Japan OAD combination | Semaglutide 0.5 mg vs OAD monotherapy + diet/exercise | ≤40 | -1.5 (-4.2 to 1.3) | 0.60 |
|  |  | >40 and ≤50 | -0.6 (-2.1 to 0.8) |  |
|  |  | >50 | -2.0 (-2.7 to -1.3) |  |
| PIONEER 1 | Semaglutide 3 mg vs Placebo | ≤40 | -1.7 (-3.5 to 0.2) | 0.37 |
|  |  | >40 and ≤50 | -0.7 (-1.7 to 0.4) |  |
|  |  | >50 | 0.0 (-0.7 to 0.7) |  |
| PIONEER 8 | Semaglutide 3 mg vs Placebo | ≤40 | -2.0 (-5.8 to 1.8) | 0.67 |
|  |  | >40 and ≤50 | -1.1 (-3.1 to 1.0) |  |
|  |  | >50 | -1.9 (-2.6 to -1.2) |  |
| PIONEER 1 | Semaglutide 7 mg vs Placebo | ≤40 | -2.8 (-4.6 to -1.0) | 0.20 |
|  |  | >40 and ≤50 | -1.4 (-2.5 to -0.2) |  |
|  |  | >50 | -1.0 (-1.7 to -0.3) |  |
| PIONEER 8 | Semaglutide 7 mg vs Placebo | ≤40 | -1.2 (-5.6 to 3.3) | 0.43 |
|  |  | >40 and ≤50 | -3.1 (-5.1 to -1.2) |  |
|  |  | >50 | -3.6 (-4.4 to -2.9) |  |
| PIONEER 4 | Semaglutide 14 mg vs Placebo | ≤40 | -1.6 (-5.2 to 2.1) | 0.53 |
|  |  | >40 and ≤50 | -2.8 (-4.5 to -1.2) |  |
|  |  | >50 | -4.0 (-4.8 to -3.1) |  |
| PIONEER 1 | Semaglutide 14 mg vs Placebo | ≤40 | -5.5 (-7.4 to -3.6) | 0.001 |
|  |  | >40 and ≤50 | -1.7 (-2.8 to -0.7) |  |
|  |  | >50 | -2.8 (-3.5 to -2.1) |  |
| PIONEER 8 | Semaglutide 14 mg vs Placebo | ≤40 | -4.9 (-9.5 to -0.3) | 0.86 |
|  |  | >40 and ≤50 | -5.4 (-7.2 to -3.5) |  |
|  |  | >50 | -4.8 (-5.5 to -4.1) |  |
| PIONEER 2 | Semaglutide 14 mg vs Empagliflozin 25 mg | ≤40 | -3.1 (-5.4 to -0.8) | 0.03 |
|  |  | >40 and ≤50 | -0.2 (-1.4 to 1.0) |  |
|  |  | >50 | -0.8 (-1.4 to -0.2) |  |
| PIONEER 4 | Semaglutide 14 mg vs Liraglutide 1.8 mg | ≤40 | -2.0 (-4.4 to 0.4) | 0.74 |
|  |  | >40 and ≤50 | -1.5 (-2.7 to -0.4) |  |
|  |  | >50 | -1.8 (-2.5 to -1.1) |  |
| PIONEER 7 | Semaglutide flex vs Sitagliptin 100 mg | ≤40 | -1.1 (-3.4 to 1.2) | 0.71 |
|  |  | >40 and ≤50 | -1.6 (-2.8 to -0.4) |  |
|  |  | >50 | -2.2 (-2.8 to -1.6) |  |

Abbreviations: CI, confidence interval; MRCT, multi-regional clinical trial; OAD, oral anti-diabetic

### Supplementary Table 7: SAEs in SUSTAIN and PIONEER programmes

| **Pool** | **Treatment** | **Age group** | **Number of participants** | **Number of participants with events** | **Proportion†** | **Rate†** |
| --- | --- | --- | --- | --- | --- | --- |
| Subcutaneous | Comparators | All participants | 3377 | 202 | 5.2 | 9.6 |
| Subcutaneous | Semaglutide | All participants | 5159 | 328 | 5.2 | 10.2 |
| Subcutaneous | Comparators | ≤40 | 274 | 13 | 4.5 | 6.2 |
| Subcutaneous | Semaglutide | ≤40 | 422 | 18 | 3.6 | 11.2 |
| Subcutaneous | Comparators | >40 - ≤50 | 701 | 30 | 4.3 | 7.0 |
| Subcutaneous | Semaglutide | >40 - ≤50 | 1041 | 59 | 5.0 | 9.7 |
| Subcutaneous | Comparators | >50 | 2402 | 159 | 5.6 | 10.7 |
| Subcutaneous | Semaglutide | >50 | 3696 | 251 | 5.5 | 10.2 |
| Oral | Comparators | All participants | 1294 | 102 | 8.2 | 11.6 |
| Oral | Semaglutide | All participants | 1912 | 129 | 8.0 | 12.8 |
| Oral | Comparators | ≤40 | 74 | 4 | 4.9 | 5.5 |
| Oral | Semaglutide | ≤40 | 110 | 4 | 4.8 | 5.1 |
| Oral | Comparators | >40 - ≤50 | 256 | 14 | 5.9 | 8.1 |
| Oral | Semaglutide | >40 - ≤50 | 363 | 14 | 3.6 | 5.4 |
| Oral | Comparators | >50 | 964 | 84 | 9.4 | 13.5 |
| Oral | Semaglutide | >50 | 1439 | 111 | 9.3 | 15.2 |

† indicates percentage of participants with events and the rate of events per 100 patient-years of exposure, which were corrected using a Cochrane-Mantel-Haenszel adjustment. The analyses for serious adverse events were performed using SAS and on-treatment data.

### Supplementary Table 8: GI SAEs in the SUSTAIN and PIONEER programmes

| **Pool**​ | **Treatment**​ | **Age group**​ | **Number of participants**​ | **Number of participants with events**​ | **Proportion†**​ | **Rate**​† |
| --- | --- | --- | --- | --- | --- | --- |
| Subcutaneous | Comparators | All participants | 3377 | 21 | 0.60 | 1.1 |
| Subcutaneous | Semaglutide​ | All participants | 5159 | 49 | 0.80 | 1.3 |
| Subcutaneous | Comparators | ≤40​ | 274​ | 2​ | 0.55​ | 0.7​ |
| Subcutaneous | Semaglutide​ | ≤40​ | 422​ | 4​ | 0.90 | 3.3​ |
| Subcutaneous | Comparators​ | >40–≤50 ​ | 701​ | 7​ | 0.97​ | 1.5​ |
| Subcutaneous | Semaglutide​ | >40–≤50 ​ | 1041​ | 11​ | 0.91​ | 1.4​ |
| Subcutaneous | Comparators | >50​ | 2402​ | 12​ | 0.5​5 | 1.1​ |
| Subcutaneous | Semaglutide​ | >50​ | 3696 | 34​ | 0.74​ | 1.1​ |
| Oral | Comparators | All participants | 1550 | 6 | 0.41 | 0.4 |
| Oral | Semaglutide​ | All participants | 2150 | 11 | 0.55 | 0.9 |
| Oral | Comparators​ | ≤40​ | 330​ | 0​ | 0​ | 0​ |
| Oral | Semaglutide​ | ≤40​ | 348​ | 0​ | 0​ | 0​ |
| Oral | Comparators​ | >40–≤50 ​ | 256​ | 1​ | 0.51​ | 0.5​ |
| Oral | Semaglutide​ | >40–≤50 ​ | 363​ | 3​ | 0.86​ | 2.6​ |
| Oral | Comparators | >50​ | 964​ | 5​ | 0.55​ | 0.5​ |
| Oral | Semaglutide​ | >50​ | 1439​ | 8​ | 0.72​ | 0.9​ |

† indicates percentage of participants with events and the rate of events per 100 patient-years of exposure, which were corrected using a Cochrane-Mantel-Haenszel adjustment. The analyses for gastrointestinal serious adverse events were performed using SAS and on-treatment data.

### Supplementary Table 9: Gastrointestinal adverse events in subcutaneously-treated participants

| **Type of AE** | **Treatment** | **Age group** | **Number of participants with events** | **Proportion†**​ | **Rate**​† |
| --- | --- | --- | --- | --- | --- |
| Total GI AEs | All comparators | ≤40 | 56 | 17.1 | 40.4 |
| Total GI AEs | All semaglutide treatment | ≤40 | 166 | 30.9 | 127.3 |
| Total GI AEs | All comparators | >40 and ≤50 | 186 | 23.5 | 62.9 |
| Total GI AEs | All semaglutide treatment | >40 and ≤50 | 429 | 33.0 | 124.9 |
| Total GI AEs | All comparators | >50 | 662 | 24.4 | 70.0 |
| Total GI AEs | All semaglutide treatment | >50 | 1,606 | 37.6 | 138.6 |
| GI AEs leading to discontinuation | All comparators | ≤40 | 2 | 0.5 | 1.1 |
| GI AEs leading to discontinuation | All semaglutide treatment | ≤40 | 9 | 1.5 | 2.8 |
| GI AEs leading to discontinuation | All comparators | >40 and ≤50 | 8 | 0.7 | 1.3 |
| GI AEs leading to discontinuation | All semaglutide treatment | >40 and ≤50 | 40 | 3.5 | 7.4 |
| GI AEs leading to discontinuation | All comparators | >50 | 41 | 1.6 | 3.4 |
| GI AEs leading to discontinuation | All semaglutide treatment | >50 | 232 | 5.7 | 11.9 |

† indicates percentage of participants with events and the rate of events per 100 patient-years of exposure, which were corrected using a Cochrane-Mantel-Haenszel adjustment. The analyses for gastrointestinal serious adverse events were performed using SAS and on-treatment data.

Abbreviations: AE, adverse event; GI, gastrointestinal.

### Supplementary Table 10: Gastrointestinal adverse events in orally-treated participants

| **Type of AE** | **Treatment** | **Age group** | **Number of participants with events** | **Proportion†**​ | **Rate**​† |
| --- | --- | --- | --- | --- | --- |
| Total GI AEs | All comparators | ≤40 | 19 | 23.1 | 50.7 |
| Total GI AEs | All semaglutide treatment | ≤40 | 31 | 36.6 | 96.3 |
| Total GI AEs | All comparators | >40 and ≤50 | 59 | 23.8 | 45.5 |
| Total GI AEs | All semaglutide treatment | >40 and ≤50 | 126 | 38.3 | 85.5 |
| Total GI AEs | All comparators | >50 | 214 | 20.1 | 38.5 |
| Total GI AEs | All semaglutide treatment | >50 | 559 | 41.6 | 96.6 |
| GI AEs leading to discontinuation | All comparators | ≤40 | 3 | 3.9 | 5.3 |
| GI AEs leading to discontinuation | All semaglutide treatment | ≤40 | 3 | 4.6 | 9.2 |
| GI AEs leading to discontinuation | All comparators | >40 and ≤50 | 6 | 2.4 | 3.7 |
| GI AEs leading to discontinuation | All semaglutide treatment | >40 and ≤50 | 16 | 3.7 | 5.1 |
| GI AEs leading to discontinuation | All comparators | >50 | 16 | 1.8 | 2.3 |
| GI AEs leading to discontinuation | All semaglutide treatment | >50 | 95 | 8.6 | 14.4 |

† indicates percentage of participants with events and the rate of events per 100 patient-years of exposure, which were corrected using a Cochrane-Mantel-Haenszel adjustment. The analyses for gastrointestinal serious adverse events were performed using SAS and on-treatment data.

Abbreviations: AE, adverse event; GI, gastrointestinal
